# Supplementary material for: Mutant KRAS-activated circATXN7 fosters tumor immunoescape by sensitizing tumor-specific T cells to activation-induced cell death
Source: Nat Commun. 2024 Jan 12;15:499. doi: 10.1038/s41467-024-44779-1 (PMC10786880; doi:10.1038/s41467-024-44779-1)
Supplement: Supplementary file 1 — Supplementary Information [file 41467_2024_44779_MOESM1_ESM.pdf]

Supplementary Information for

Mutant KRAS-activated circATXN7 fosters tumor immunoescape by sensitizing tumor-specific T cells to activation-induced cell death

Author: Chi Zhou, Wenxin Li, Zhenxing Liang, Xianrui Wu, Sijing Cheng, Jianhong Peng, Kaixuan Zeng, Weihao Li, Ping Lan, Xin Yang, Li Xiong, Ziwei Zeng, Xiaobin Zheng, Liang Huang, Wenhua Fan, Zhanzhen Liu, Yue Xing, Liang Kang, Huashan Liu

Correspondence:

1. Huashan Liu, MD, PhD; Email: liuhshan@mail2.sysu.edu.cn.
2. Liang Kang, MD, PhD; Email: kangl@mail.sysu.edu.cn
3. Yue Xing, MD, PhD; Email: xingy28@mail.sysu.edu.cn

This File Includes:

1. Supplementary Fig. 1-14
2. Supplementary Table 1-2
3. References

Supplementary Figures and Legends

Supplementary Fig. 1

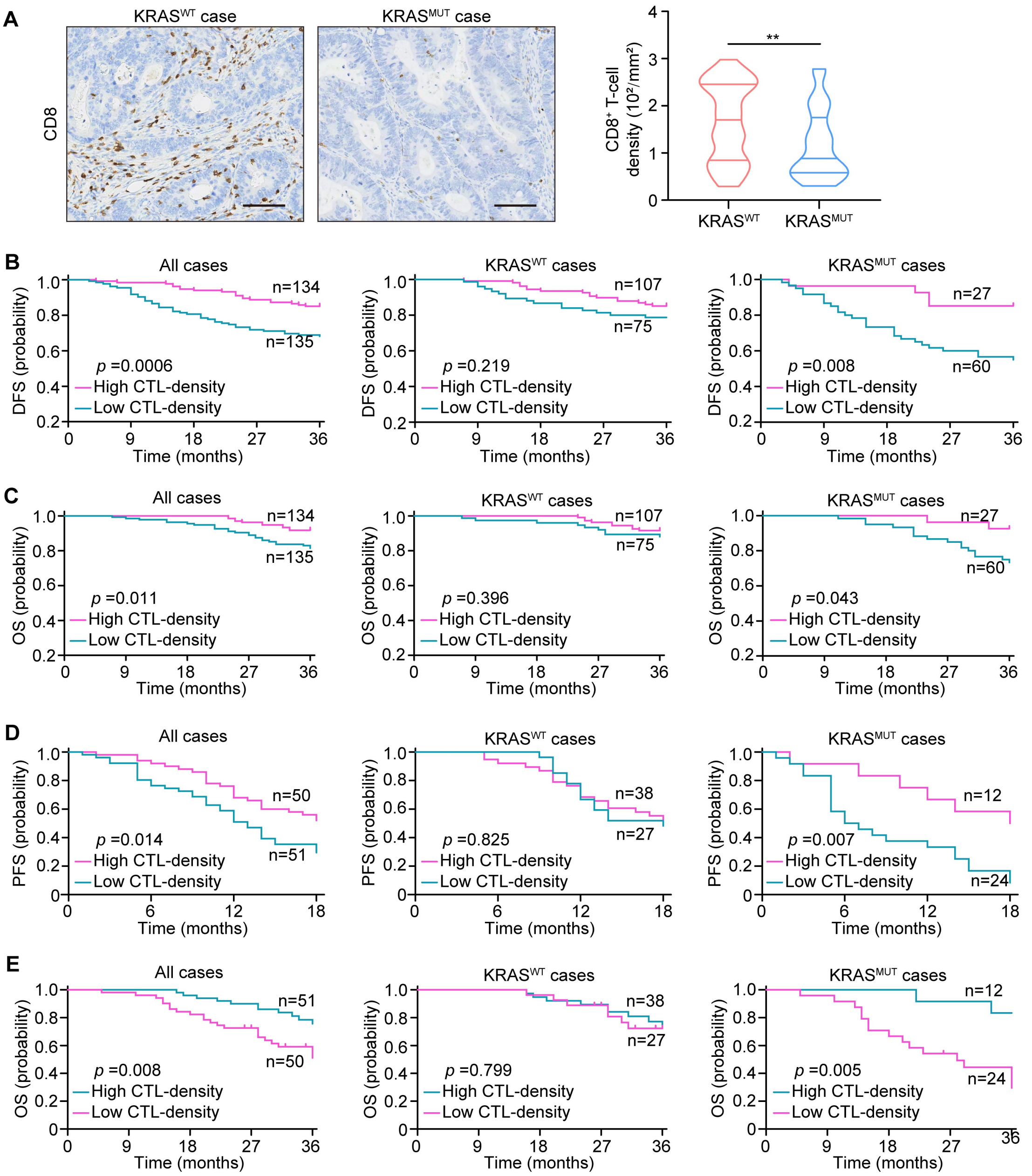

Supplementary Fig. 1 related to Fig. 1. (**A**) Immunostaining and quantification of $CD8^+$ T cells in $KRAS^{WT}$ (n = 65 patients) and $KRAS^{MUT}$ (n = 36 patients) CRC tissues from SYSUCC. Scale bars: 100 μm. (**B-C**) Kaplan–Meier survival curves for DFS (**B**) and OS (**C**) layered by CTL-density in $KRAS^{WT}$ (n = 182 patients) and $KRAS^{MUT}$ (n = 87 patients) CRC cases from SYSU-6thAH. (**D-E**) Kaplan–Meier curves for PFS (**D**) and OS (**E**) layered by CTL-density in $KRAS^{WT}$ (n = 65 patients) and $KRAS^{MUT}$ (n = 36 patients) CRC patients from SYSUCC. $^{**}p \leq 0.01$ by two-

sided Mann-Whitney test (**A**) or two-sided log-rank test (**B**-**E**). Source data and exact p values are provided as a Source Data file.

Supplementary Fig. 2

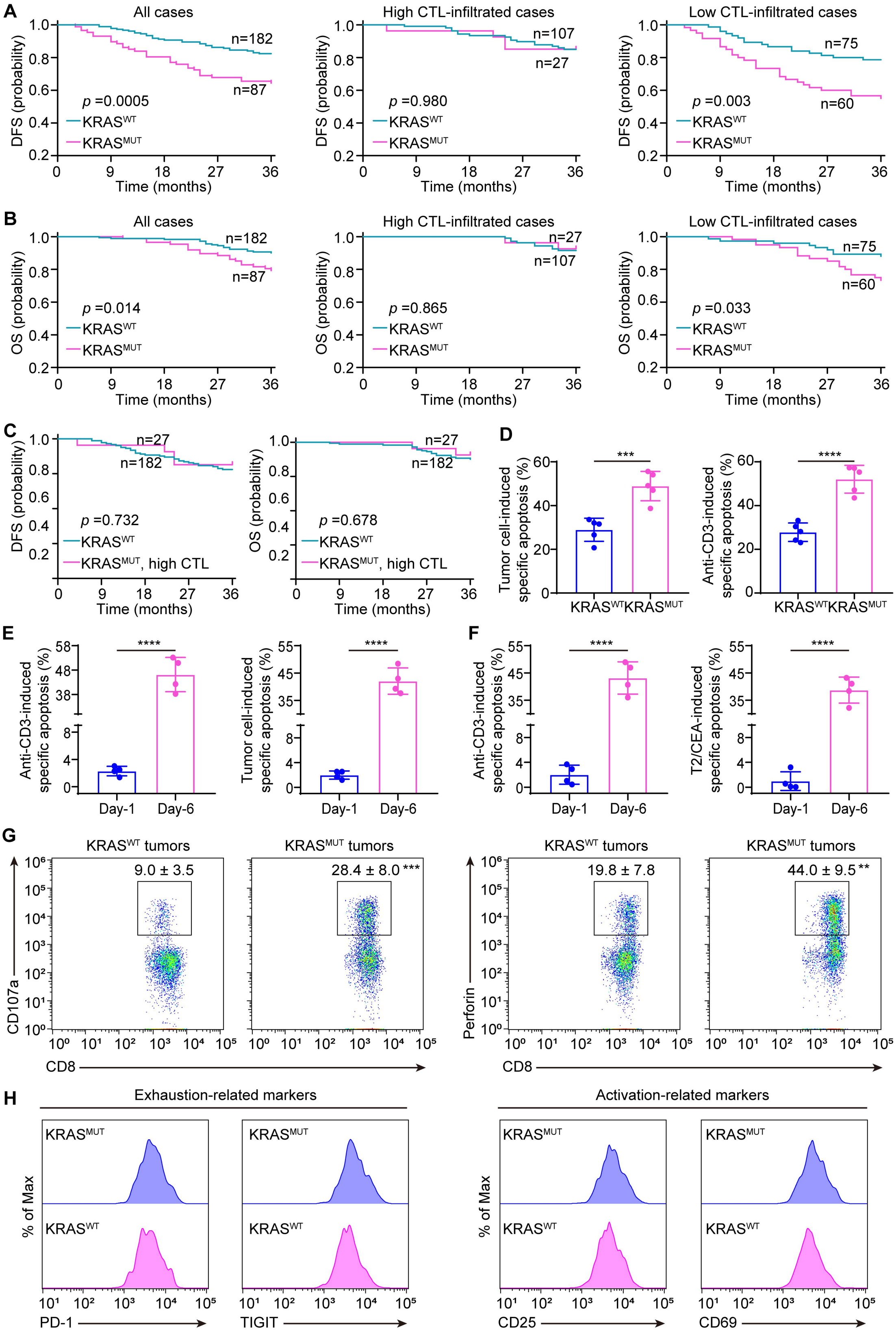

Supplementary Fig. 2 related to Fig. 1. Kaplan–Meier curves for (**A**) DFS and (**B**) OS layered by KRAS status in high (n = 134 patients) and low (n = 135 patients)

CTL-infiltrated cases from SYSU-6thAH. (**C**) Kaplan-Meier curves for DFS and OS for $KRAS^{WT}$ patients (n = 182 patients) and high CTL-infiltrated $KRAS^{MUT}$ patients (n = 27 patients) from SYSU-6thAH. (**D**) Graph for **Fig. 1A** showing statistical comparison of tumor cell- or anti-CD3-induced specific apoptosis of CTLs from stage IV CRCs with the indicated KRAS status (n = 5 samples). (**E**) Graph for **Fig. 1C** showing statistical comparison of anti-CD3- or tumor cell-induced specific apoptosis of tumor-antigen activated CTLs (n = 4 samples). (**F**) Graph for **Fig. 1D** showing statistical comparison of anti-CD3- or T2/CEA-induced specific apoptosis of CEA activated CTLs (n = 4 samples). (**G-H**) Tumor-specific CTLs were freshly isolated from $KRAS^{MUT}$ versus $KRAS^{WT}$ CRCs. (**G**) Surface marker CD107a and Intracellular perforin were analyzed by flow cytometry (n = 5 samples). Numbers (mean ± SD) indicate the percentages of gated cells stained for perforin, or CD107a (*p* value was for comparison with $KRAS^{WT}$). (**H**) Flow cytometry showing the surface marker (PD-1, TIGIT, CD25, and CD69) in the indicated CTLs (n = 5 samples). Statistical data presented in this figure show mean values ± SD (**D-F**). $^{**}p \leq 0.01$, $^{***}p \leq 0.001$ and $^{***}p \leq 0.0001$, by two-sided log-rank test (**A-C**), two-tailed Student's t-test (**D-G**). Source data and exact p values are provided as a Source Data file.

Supplementary Fig. 3

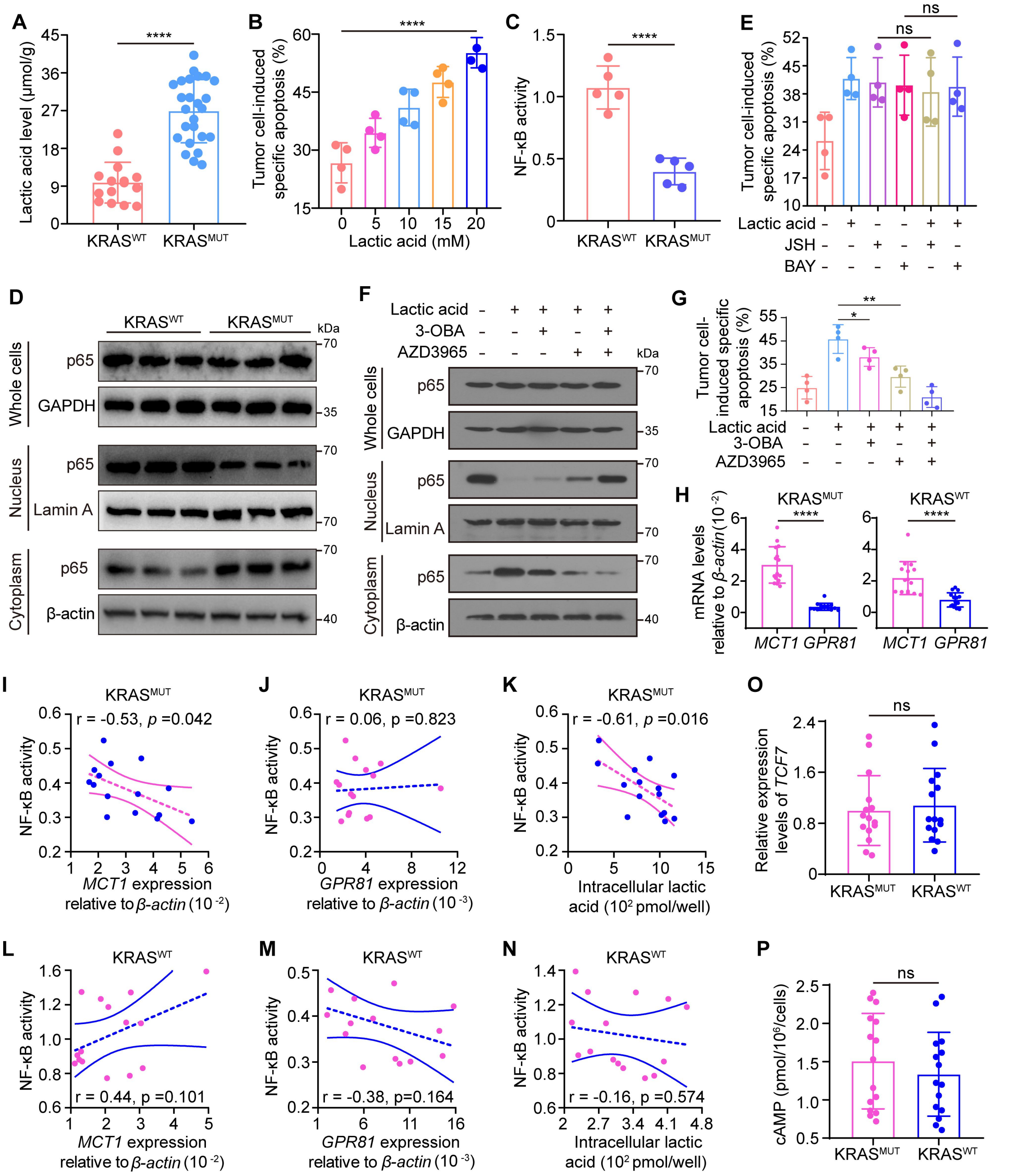

Supplementary Fig. 3 related to Fig. 2. (**A**) Lactic acid levels in KRAS^{WT} (n = 15 patients) versus KRAS^{MUT} (n = 25 patients) stage IV CRCs. (**B**) Tumor-specific CTLs from KRAS^{WT} stage IV CRCs were pretreated with indicated concentrations of lactic acid for 12 h, and then subjected to autologous tumor cell stimulation. The plots represent tumor cell-induced specific apoptosis (n = 4 samples). (**C**) NF-κB activity in tumor-specific CTLs from KRAS^{WT} versus KRAS^{MUT} stage IV CRCs (n = 5 samples). (**D**) Western blots showing p65 localization in tumor-specific CTLs from KRAS^{WT}

versus $KRAS^{MUT}$ stage IV CRCs (n = 3 samples). (**E**) Statistical comparison of autologous tumor cell-induced specific apoptosis of $KRAS^{WT}$ stage IV CRC-derived tumor-specific CTLs pretreated with JSH or BAY in combination with PBS or 10 mM lactic acid (n = 4 samples). (**F**) Western blots showing NF-κB activity in $KRAS^{WT}$ CRC-derived tumor-specific CTLs pretreated with AZD3965 or 3-OBA in combination with PBS or lactic acid. Three independent experiments were performed and similar results were obtained. Lamin A and β-actin served as nuclear and cytoplasmic loading controls, respectively. (**G**) Statistics of autologous tumor cell-induced specific apoptosis of $KRAS^{WT}$ CRC-derived tumor-specific CTLs pretreated with AZD3965 or 3-OBA in combination with PBS or 10 mM lactic acid (n = 4 samples). (**H**) qRT-PCR showing expression of *MCT1* and *GPR81* in the tumor-specific CTLs of $KRAS^{MUT}$ or $KRAS^{WT}$ tumors (n = 15 samples). (**I**) Correlation between NF-κB activity and MCT1 expression in tumor-specific CTLs from $KRAS^{MUT}$ CRCs (n = 15 samples). (**J**) Correlation between NF-κB activity and *GPR81* expression in tumor-specific CTLs from $KRAS^{MUT}$ CRCs (n = 15 samples). (**K**) Correlation between NF-κB activity and intracellular lactic acid concentration in tumor-specific CTLs from $KRAS^{MUT}$ CRCs (n = 15 samples). (**L**) Correlation between NF-κB activity and *MCT1* expression in tumor-specific CTLs from $KRAS^{WT}$ CRCs (n = 15 samples). (**M**) Correlation between NF-κB activity and *GPR81* expression in tumor-specific CTLs from $KRAS^{WT}$ CRCs (n = 15 samples). (**N**) Correlation between NF-κB activity and intracellular lactic acid concentration in tumor-specific CTLs from $KRAS^{WT}$ CRCs (n = 15 samples). (**O**) qRT-PCR showing *TCF-1* expression in the tumor-specific CTLs of $KRAS^{MUT}$ versus $KRAS^{WT}$ tumors (n = 15 samples). (**P**) cAMP levels in the tumor-specific CTLs of $KRAS^{MUT}$ versus $KRAS^{WT}$ tumors (n = 15 samples). Statistical data presented in this figure show mean values ± SD (**A-C, E, G-H, O-P**). $^{*}p \leq 0.05$, $^{**}p \leq 0.01$, $^{****}p \leq 0.0001$ and ns indicates $p > 0.05$, by two-tailed Student's t-test (**A**, **C**, **H**, **O-P**), one-way ANOVA (**B**, **E**, **G**), and Pearman correlation (**I-N**). Source data and exact p values are provided as a Source Data file.

Supplementary Fig. 4

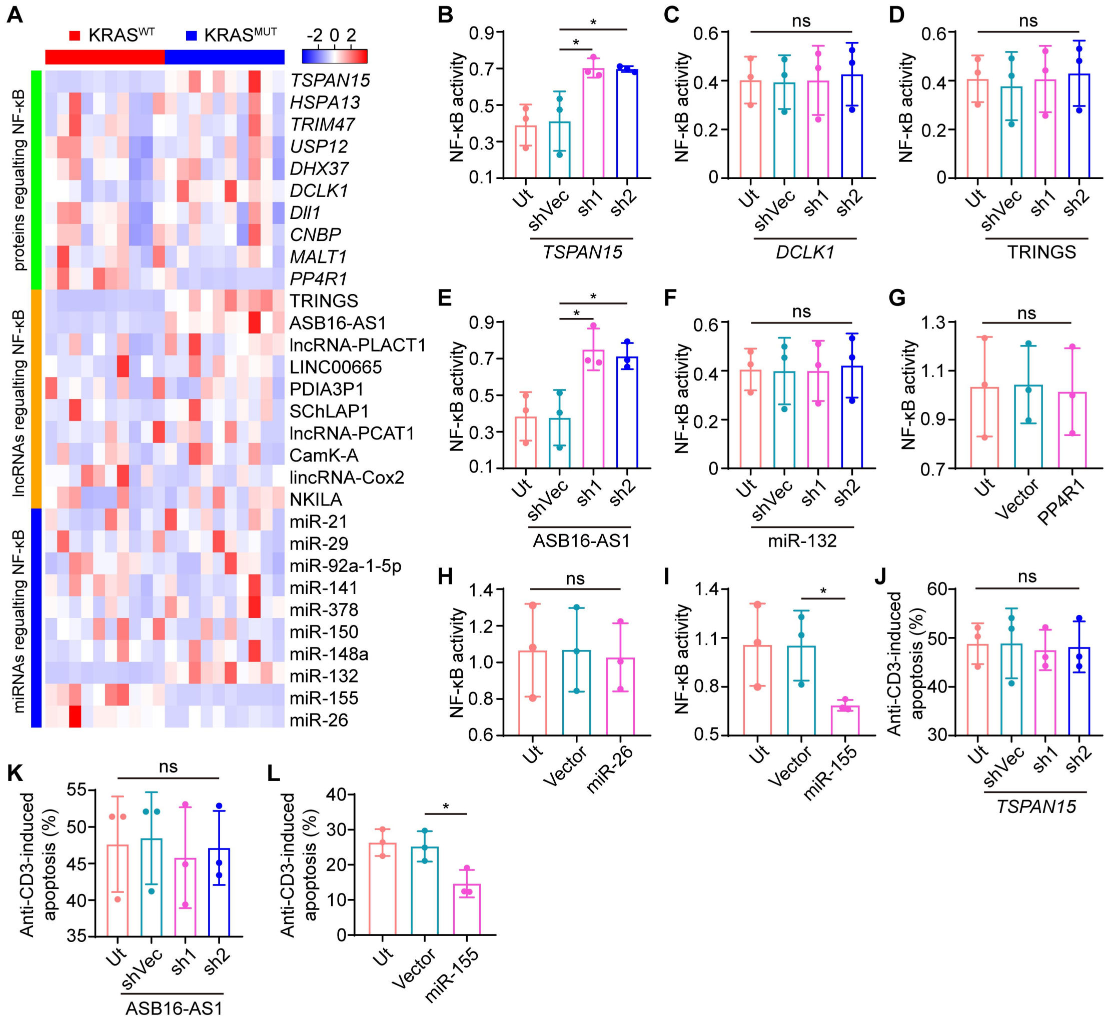

Supplementary Fig. 4 related to Fig. 2. (**A**) Heatmap showing the expression levels of the published factors regulating NF-κB in tumor-specific CTLs of KRAS^{MUT} versus KRAS^{WT} tumors (n = 10 samples). (**B-F**) NF-κB activity in KRAS^{MUT} CRCs-derived tumor-specific CTLs transduced with lentivirus carrying an expression cassette for the shRNAs targeting the indicated molecules (sh1 or sh2) or shRNA control vector (shVec) (n = 3 samples). Ut, KRAS^{MUT} CRCs-derived tumor-specific CTLs without any treatment. (**G-I**) NF-κB activity in KRAS^{WT} CRCs-derived tumor-specific CTLs with overexpression of the indicated molecules or control vector (Vector) (n = 3 samples). Ut, KRAS^{WT} CRCs-derived tumor-specific CTLs without any treatment. (**J-L**) Apoptosis of the indicated tumor-specific CTLs induced by anti-CD3 (n = 3 samples). Ut, cells without any treatment. Statistical data presented in this

figure show mean values ± SD (**B-L**). *$p \leq 0.05$, and ns indicates $p > 0.05$, by one-way ANOVA (**B-L**). Source data and exact p values are provided as a Source Data file.

Supplementary Fig. 5

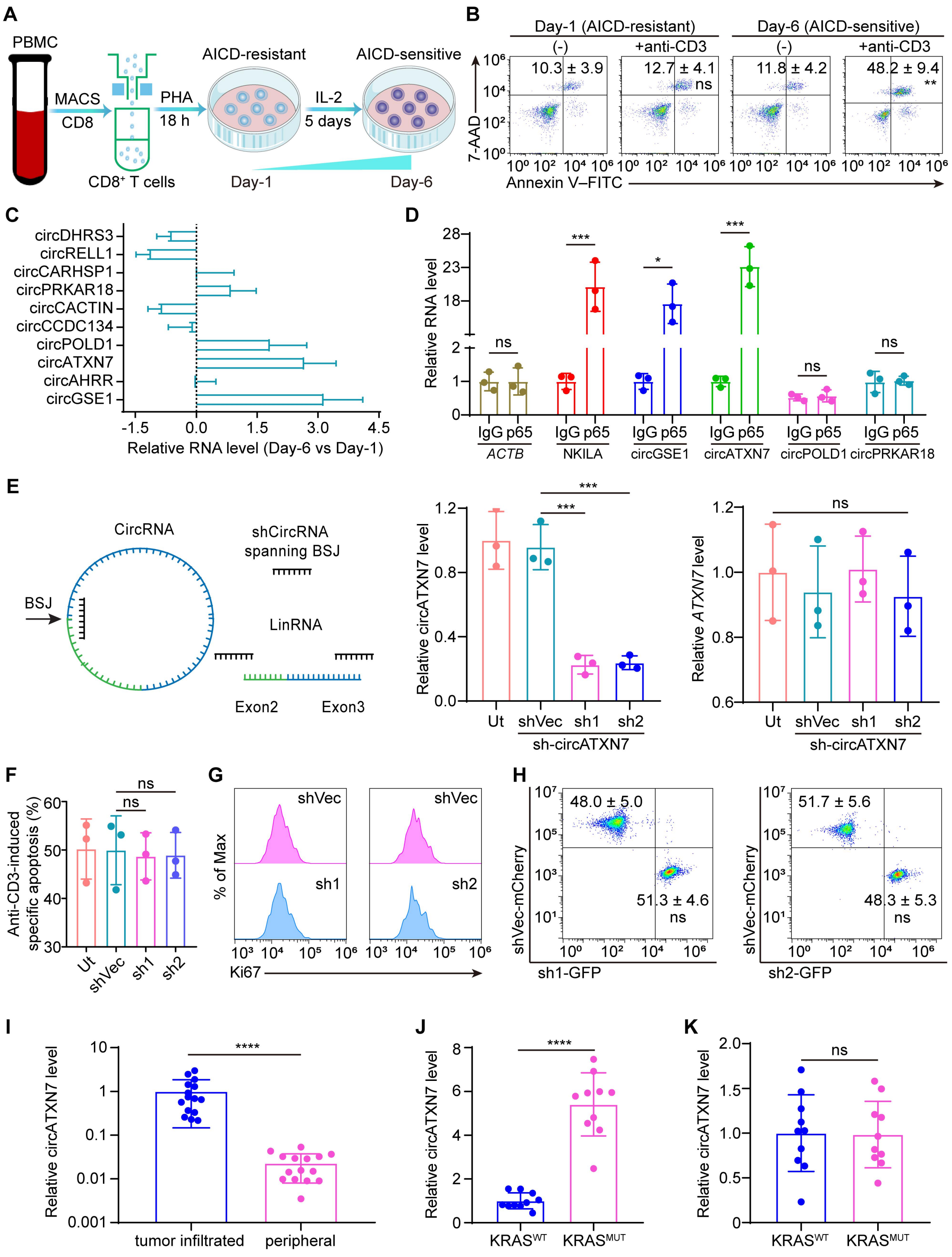

Supplementary Fig. 5 related to Fig. 2. (**A**) Schematic diagram showing *ex vivo* AICD induction in peripheral CD8⁺ T cells from healthy donors. (**B**) Representative apoptosis plots of the indicated cells induced by anti-CD3 (n = 3 independent samples). Numerical values (mean ± SD) denote annexin V⁺ cell percentages (*p* values were for comparison with the untreated cells). (**C**) Expression levels of the

indicated circRNAs in Day 6 versus Day 1 T cells (n = 3 independent samples). (**D**) RIP assay in Day 6 T cells using an antibody against p65, followed by detection of the indicated circRNAs (n = 3 independent samples). Values were normalized to the background RIP level, as detected by an IgG isotype control. *ACTB* and *NKILA* serve as negative and positive controls, respectively. (**E**) Expression measured by qRT-PCR of the linear transcripts after targeting of the circular isoforms by using shRNAs (sh1 or sh2) against the backsplice junction (BSJ). Control vector (shVec) was used as controls (n = 3 independent samples). Ut, Day-6 T cells without any treatment. (**F**) Statistics of anti-CD3-induced specific apoptosis of Day-6 T cells transduced with lentivirus carrying an expression cassette for circGSE1 shRNA (sh1 or sh2) or shRNA control vector (shVec) (n = 3 independent samples). (**G**) Flow cytometric quantification of Ki-67 expression showing no effects of circATXN7 knockdown on Day-6 T cell proliferation (n = 3 independent samples) (**H**) Transwell assay-flow cytometry analysis showing similar migratory abilities of Day-6 T cells with or without circATXN7 knockdown (n = 5 independent samples; *p* values were for comparison with shVec). (**I**) Statistical comparison of circATXN7 expression in tumor infiltrated versus peripheral $CD8^+$ T cells (n = 15 independent samples). (**J**) qRT-PCR showing expression levels of circATXN7 in tumor-specific CTLs derived from $KRAS^{MUT}$ versus $KRAS^{WT}$ CRC tissues (n = 10 samples). (**K**) qRT-PCR showing expression levels of circATXN7 in tumor non-specific CTLs derived from $KRAS^{MUT}$ versus $KRAS^{WT}$ CRC tissues (n = 10 samples). Statistical data presented in this figure show mean values ± SD (**D-F, I-K**). *$p \leq 0.05$, ***$p \leq 0.001$, ****$p \leq 0.0001$, and ns indicates $p > 0.05$, by two-tailed Student's t-test (**B**, **D**, **H**, **I-K**) or one-way ANOVA (**E-F**). Source data and exact p values are provided as a Source Data file.

Supplementary Fig. 6

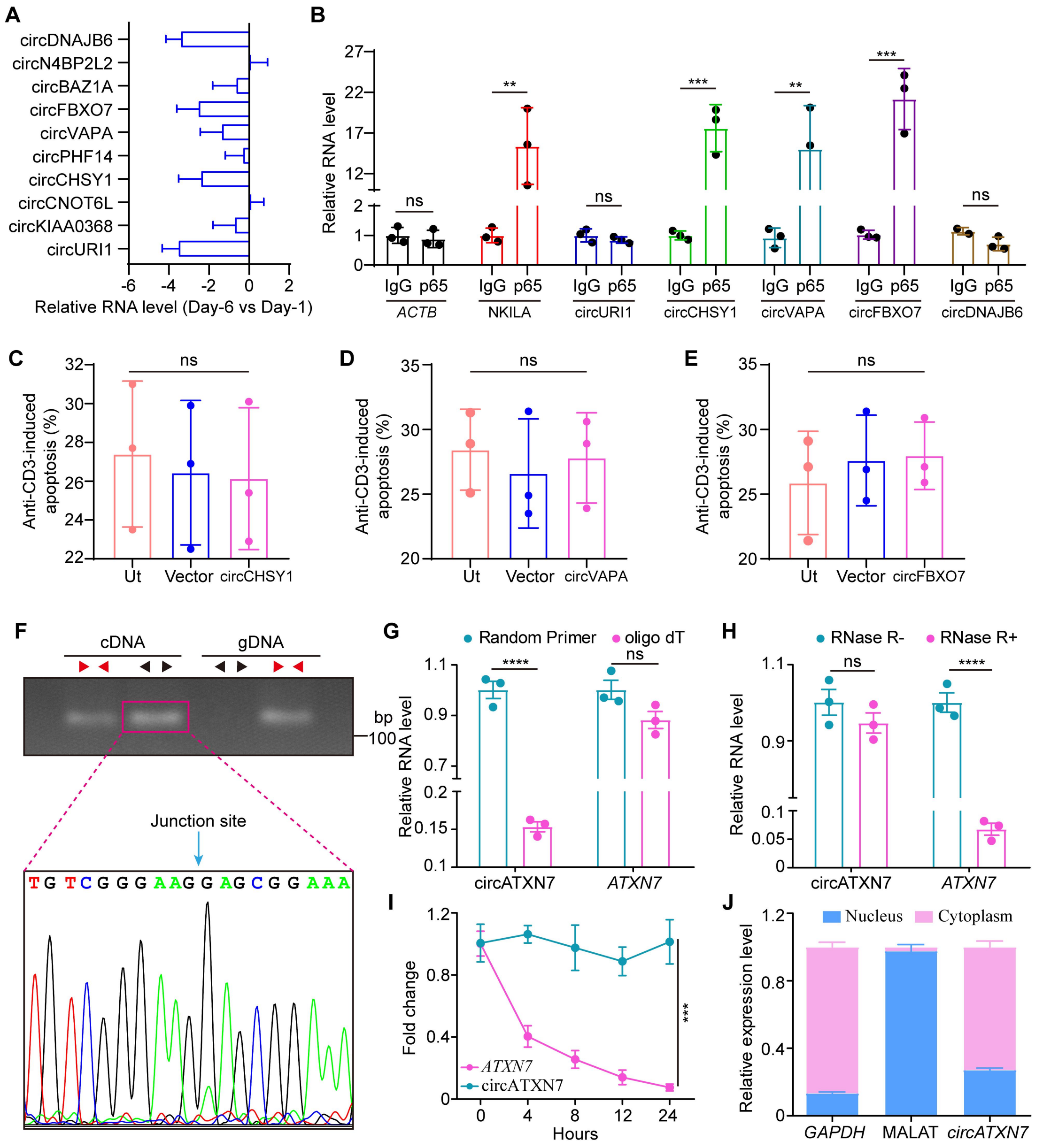

Supplementary Fig. 6 related to Fig. 2. (**A**) Expression levels of the indicated circRNAs in Day 6 versus Day 1 T cells (n = 10 independent samples). (**B**) RIP assay in Day 6 T cells using an antibody against p65, followed by detection of the indicated circRNAs (n = 3 independent samples). Values were normalized to the background RIP level, as detected by an IgG isotype control. *ACTB* and *NKILA* serve as negative and positive controls, respectively. (**C-E**) Anti-CD3-induced apoptosis of the Day 1 T cells with overexpression of the indicated circRNAs or control vector (Vector) (n = 3 independent samples). Ut, KRAS^{WT} CRCs-derived tumor-specific CTLs without any

treatment. (**F**) RT-PCR for the analysis of circATXN7 existence in Day-6 T cells using the divergent primers and convergent primers. Sanger sequencing confirmed *ATXN7* back-splicing. (**G**) qRT-PCR for circATXN7 and *ATXN7* linear mRNA in oligo dT constructed cDNA or random primers constructed cDNA (n = 3 independent samples). (**H**) qRT-PCR for circATXN7 and *ATXN7* linear mRNA upon RNase R treatment (n = 3 independent samples). (**I**) qRT-PCR for circATXN7 and *ATXN7* linear mRNA abundance in Day-6 T cells treated with actinomycin D at the indicated time points (n = 3 independent samples). **(J)** Cytoplasmic and nuclear mRNA fractionation experiment in Day-6 T cells (n = 3 independent samples). *GADPH* and *MALAT1* served as markers of cytoplasmic and nuclear locations, respectively. Statistical data presented in this figure show mean values ± SD (**A-E, G-J**). $^{**}p \leq 0.01$, $^{***}p \leq 0.001$, $^{****}p \leq 0.0001$ and ns indicates $p > 0.05$, by two-tailed Student's t-test (**B**, **I**) or one-way ANOVA (**C-E**, **G-H**). Source data and exact p values are provided as a Source Data file.

Supplementary Fig. 7

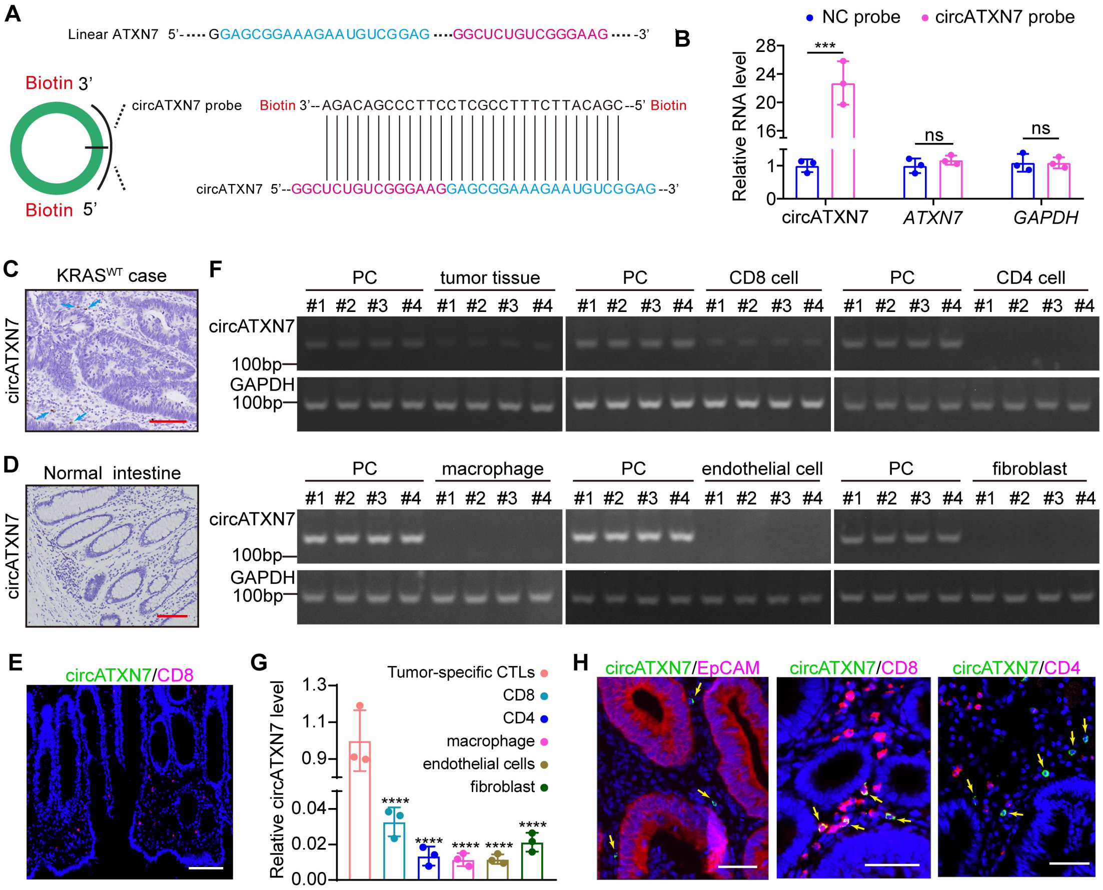

Supplementary Fig. 7 related to Fig. 3. (**A**) The design of the probe that specifically detects circATXN7 but not the linear one. (**B**) RNA pulldown assay in Day 6 T cells using the circATXN7 probe, followed by detection of the enrichment of circATXN7, *ATXN7* mRNA, and *GAPDH* mRNA (n = 3 independent samples). (**C**) Representative circATXN7 ISH staining in KRAS^{WT} CRC cases (n = 3 patients). Scale bar: 100 µm. (**D**) circATXN7 ISH staining in benign intestine. Scale bar: 100 µm. (**E**) Representative images for circATXN7 fluorescence in situ hybridization (green) and CD8 (red) co-staining in normal adjacent tissues (n = 3 patients). Scale bars: 100 µm. (**F**) RT-PCR analysis of circATXN7 expression in tumor tissues as well as CD8^{+} cells, CD4^{+} cells, macrophages, endothelial cells, and fibroblasts purified from fresh CRC tissues (n = 4 patients). Day 6 T cells served as positive control (PC). *GAPDH* served as loading control. (**G**) qRT-PCR showing circATXN7 expression levels in the indicated cells purified from CRC tissues (n=3 samples; *p* values were for comparison with the tumor-specific CD8^{+} cells). (**H**) Representative images for circATXN7

fluorescence in situ hybridization (green) and EpCAM (red), CD8 (red), or CD4 (red) co-staining in CRC tissues (n = 3 patients). Scale bars: 50 μm. Statistical data presented in this figure show mean values ± SD (**B**, **G**). ***$p \leq 0.001$, ****$p \leq 0.0001$ and ns indicates $p > 0.05$, by two-tailed Student's t-test (**B**, **G**). Source data and exact p values are provided as a Source Data file.

Supplementary Fig. 8

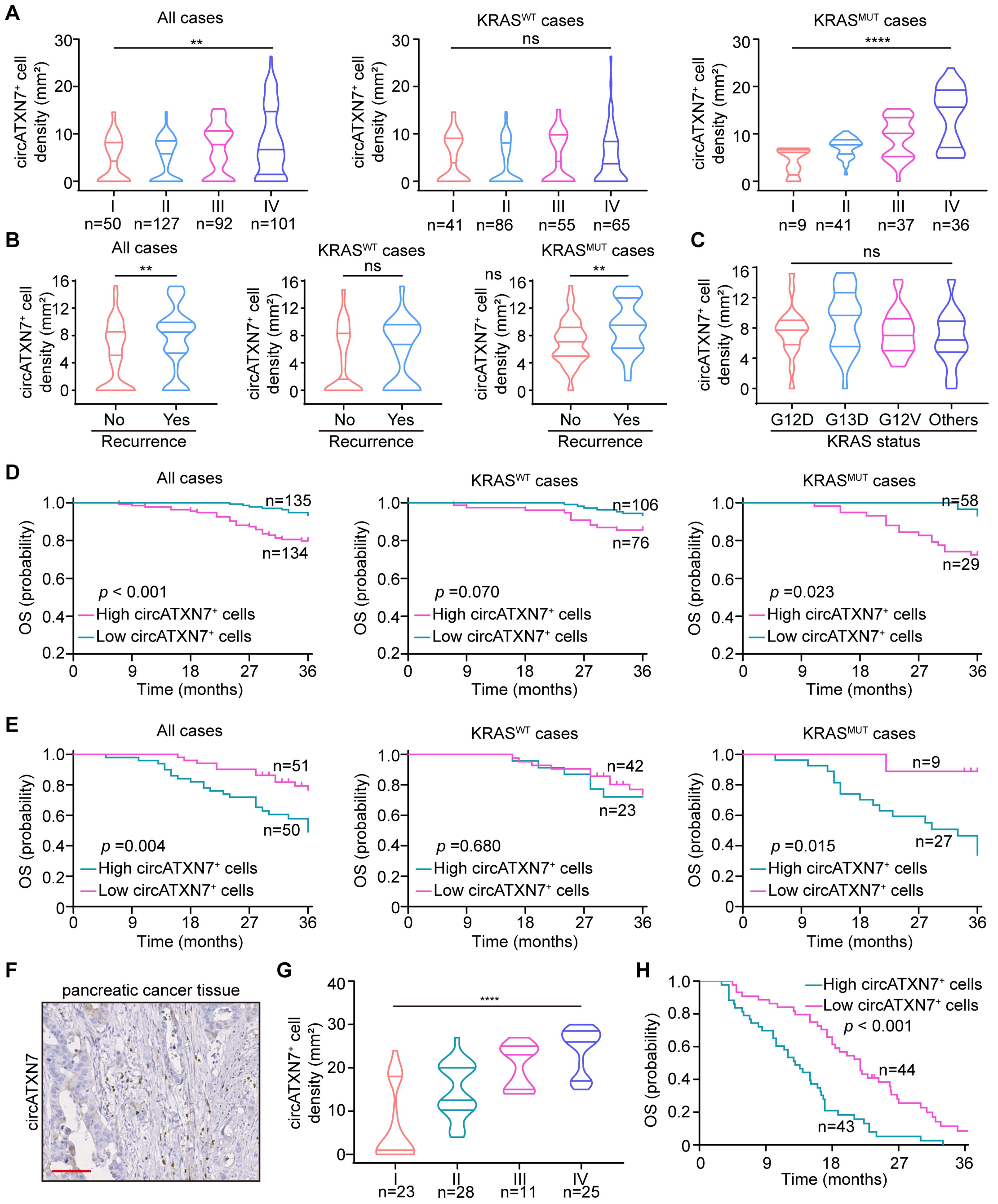

Supplementary Fig. 8 related to Fig. 3. (**A-B**) Statistics of circATXN7^{+} cell density in CRCs with different TNM stages (**A**) and in patients with or without recurrence at 3 years (**B**). (**C**) Statistics of circATXN7^{+} cell density in CRCs with the indicated KRAS mutations. (**D-E**) Kaplan–Meier curves for OS layered by circATXN7^{+} cell density in 269 CRC cases from SYSU-6thAH (**D**) and in 101 patients from SYSUCC (**E**). (**F**) Representative circATXN7 ISH staining in pancreatic cancer cases (n = 87

patients). Scale bar: 100 µm. (**G**) Statistics of circATXN7$^+$ cell density in pancreatic cancers with different TNM stages (n = 87 patients). (**H**) Kaplan–Meier survival curves for OS layered by circATXN7$^+$ cell density in patients with pancreatic cancers (n = 87 patients). Statistical data presented in this figure show mean values ± SD (**A-C**, **G**). $^{**}p \leq 0.01$, $^{****}p \leq 0.0001$, and ns indicates $p > 0.05$, by two-sided Kruskal-Wallis H test (**A**, **G**), two-sided Mann-Whitney test (left and middle panels of **B**), two-tailed Student's t-test (right panel of **B**), 1-way ANOVA (**C**), or two-sided log-rank test (**D-E**, and **H**). Source data and exact p values are provided as a Source Data file.

Supplementary Fig. 9

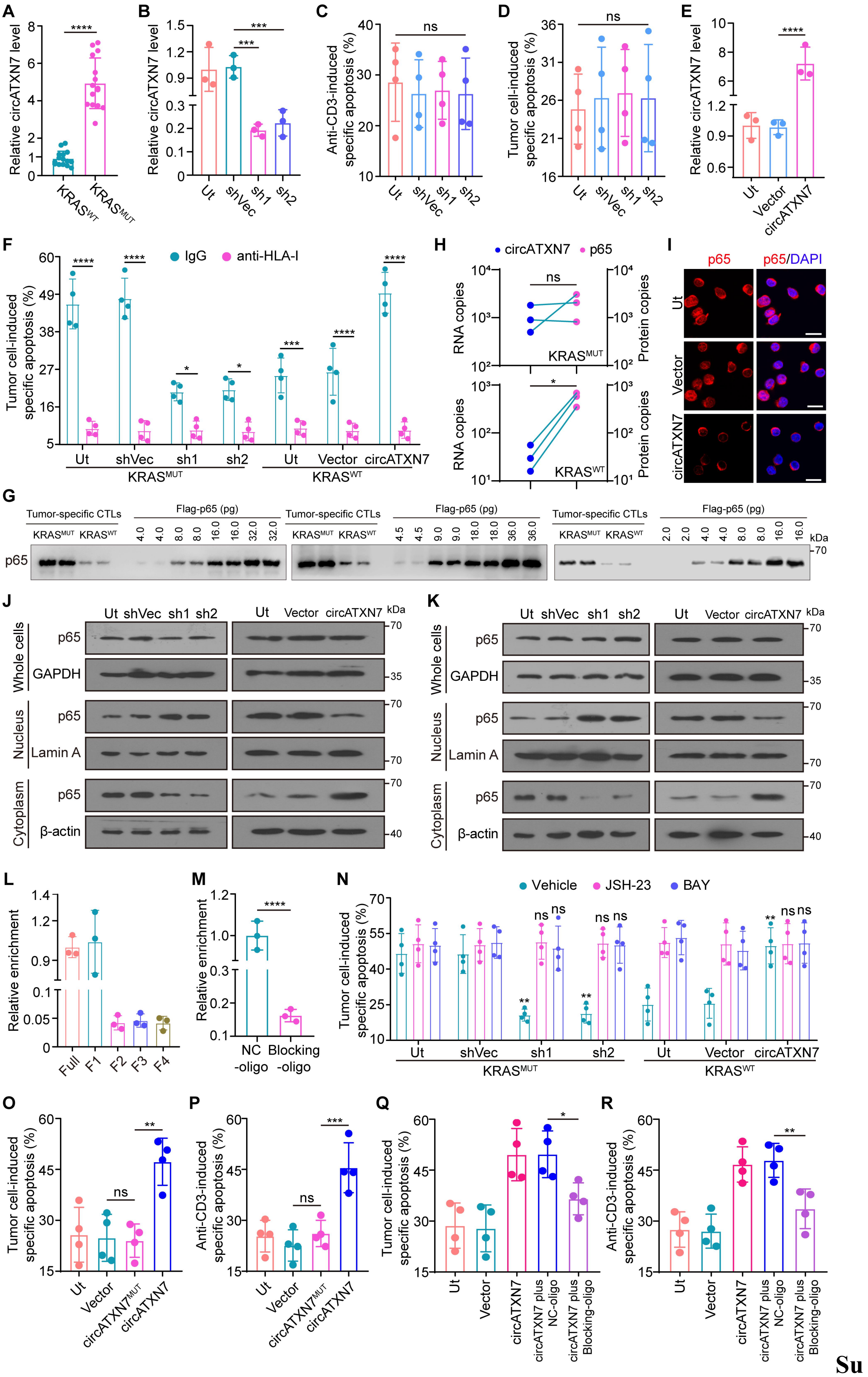

Su

pplementary Fig. 9 related to Fig. 4. (**A**) circATXN7 expression levels in tumor-specific CTLs from $KRAS^{WT}$ versus $KRAS^{MUT}$ CRCs (n = 15 patients). (**B**) circATXN7 expression levels in $KRAS^{MUT}$ CRC-derived tumor-specific CTLs transduced with lentivirus carrying an expression cassette for circATXN7 shRNA (sh1 or sh2) or shRNA control vector (shVec) (n = 3 independent samples). (**C-D**) Statistics of anti-CD3- (**C**) or autologous tumor cell-induced (**D**) specific apoptosis of $KRAS^{WT}$ tumor-derived tumor-specific CTLs with circATXN7 sh1, sh2, or shVec (n = 4 independent samples). (**E**) circATXN7 expression levels in tumor-specific CTLs from $KRAS^{WT}$ CRCs with circATXN7 overexpression or control vector (Vector) (n = 3 samples). (**F**) Autologous tumor cell-induced specific apoptosis of indicated tumor-specific CTLs preincubated with anti-HLA-I or IgG (n = 4 samples). (**G**) Measurement of the copy number of p65 protein in the cytoplasm of tumor-specific CTLs of KRASWT and KRASMUT tumors. Purified recombinant p65 protein was used to generate standard curves to estimate the mass of p65 in cytoplasmic extracts of 1×10^5 tumor-specific CTLs. (**H**) Statistics of circATXN7 and p65 copy number in the cytoplasm of tumor-specific CTLs of $KRAS^{WT}$ and $KRAS^{MUT}$ tumors. (**I**) IF staining for p65 (red) nuclear translocation in $KRAS^{WT}$ tumor-derived tumor-specific CTLs with circATXN7 overexpression or Vector (n = 3 samples). Scale bars: 10 μm. (**J**) p65 nuclear translocation in $KRAS^{MUT}$ CRC-derived tumor-specific CTLs with circATXN7 sh1, sh2, or shVec (left panel), or in $KRAS^{WT}$ CRC-derived tumor-specific CTLs with circATXN7 overexpression or Vector (right panel). (**K**) p65 nuclear translocation in Day 6 T cells with circATXN7 sh1, sh2, or shVec (left panel), or in Day 1 T cells with circATXN7 overexpression or Vector (right panel). (**L**) RIP assay using an antibody against Flag in lysates prepared from Day 6 T cells transfected with full-length p65 or its respective deletion mutants (n = 3 independent samples). (**M**) RIP assay using an antibody against Flag in lysates prepared from Day 6 T cells treated with NC or blocking oligos (n = 3 independent samples). Values were normalized to the background RIP level, as detected by an IgG isotype control. (**N**) Autologous tumor cell-induced specific apoptosis in $KRAS^{MUT}$ CRC-derived tumor-

specific CTLs with circATXN7 sh1, sh2, or shVec, or in $KRAS^{WT}$ CRC-derived tumor-specific CTLs with or without circATXN7 overexpression, in combination with Vehicle, JSH-23 (6 mM) or BAY (2 mM) (n = 4 independent samples). (**O**) Autologous tumor cell-induced specific apoptosis of $KRAS^{WT}$ CRC-derived tumor-specific CTLs transduced with lentivirus carrying an expression cassette for circATXN7, binding site-mutated circATXN7 ($circATXN7^{MUT}$), or Vector (n = 4 samples). (**P**) Anti-CD3-induced specific apoptosis in Day 1 T cells with overexpression of circATXN7, $circATXN7^{MUT}$, or Vector (n = 4 independent samples). (**Q**) Autologous tumor cell-induced specific apoptosis of $KRAS^{WT}$ CRC-derived tumor-specific CTLs transduced with lentivirus carrying an expression cassette for circATXN7, in combination with NC or blocking oligos (n = 4 samples). (**R**) Anti-CD3-induced specific apoptosis of Day 1 T cells with circATXN7 overexpression, in combination with NC or blocking oligos (n = 4 independent samples). Statistical data presented in this figure show mean values ± SD (**A-F, L-R**). Ut, cells without any treatment (**B**-**F**, **I**-**K**, and **N**-**R**). In panels G, J-K, three independent experiments were performed and similar results were obtained. *$p \leq 0.05$, **$p \leq 0.01$, ***$p \leq 0.001$, ****$p \leq 0.0001$ and ns indicates $p > 0.05$, by two-tailed Student's t-test (**A** and **M**), paired t-test (H) or one-way ANOVA (**B**-**F**, **L**, and **N**-**R**). Source data and exact p values are provided as a Source Data file.

Supplementary Fig. 10

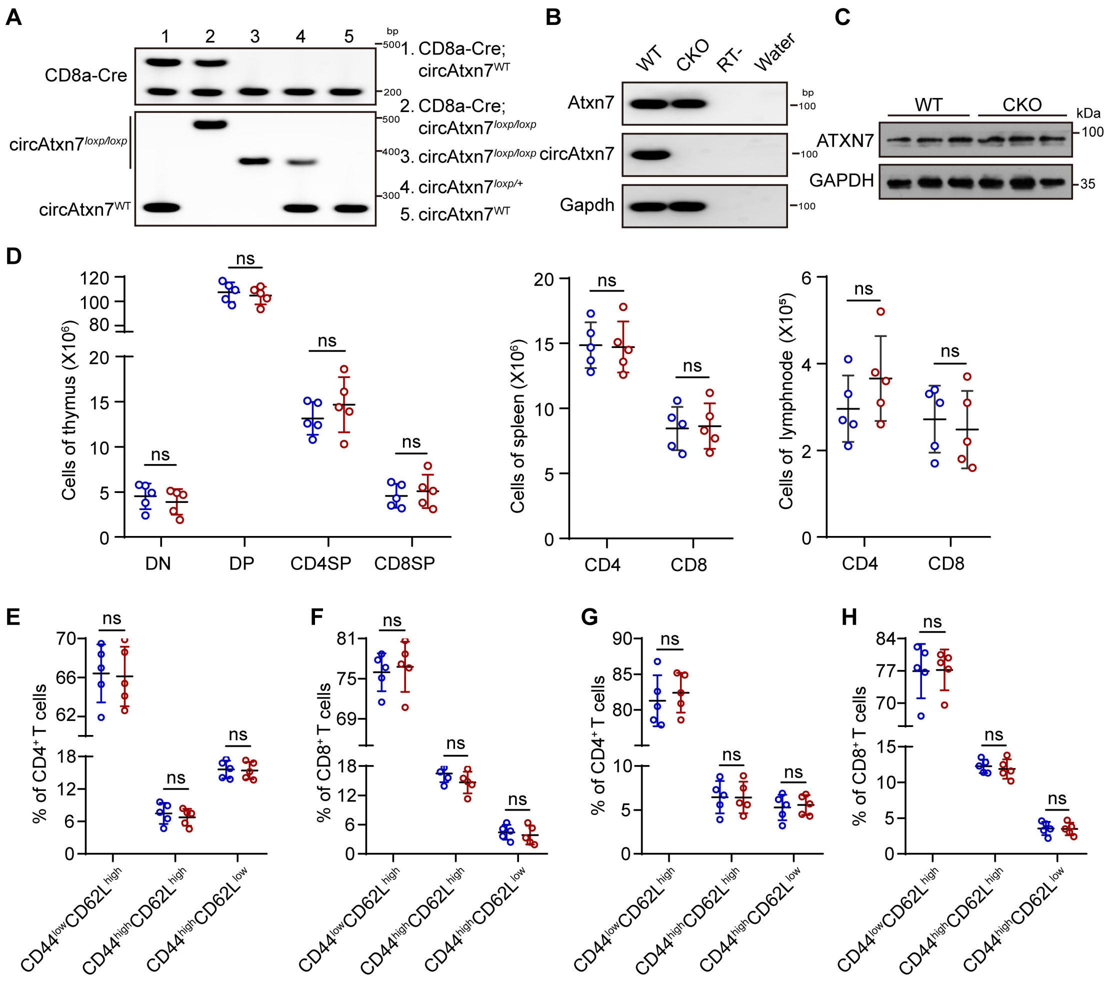

Supplementary Fig. 10 related to Fig. 5. (**A**) PCR genotyping for the indicated mice. (**B**) RNA level in spleen CD8$^+$ T cells of *circAtxn7^{CKO}* (termed CKO) or wild-type (WT) mice. (**C**) Western blot showing the expression levels of ATXN7 protein in spleen CD8$^+$ T cells of CKO or WT mice (n = 3 animals). (**D**) Numbers of CD4$^-$CD8$^-$ double-negative (DN), CD4$^+$CD8$^+$ double-positive (DP), CD4$^+$CD8$^-$ (CD4 single-positive, CD4SP), CD4$^-$CD8$^+$ (CD8 single-positive, CD8SP) cells in the thymus, as well as CD4$^+$ and CD8$^+$ T cells in the lymphnodes and spleens of 8-week-old WT and CKO mice (n = 5 animals). (**E-H**) Percentages of CD44^{lo}CD62L^{hi}, CD44^{hi}CD62L^{hi}, and CD44^{hi}CD62L^{lo} subtypes for CD4$^+$ and CD8$^+$ T cells in the lymphnodes (**E-F**) and spleens (**G-H**) of WT and CKO mice (n = 5 animals). Statistical data presented in this figure show mean values ± SD (**D-H**). In panels A-B, three independent experiments were performed and similar results were obtained. ns indicates $p > 0.05$,

by two-tailed Student's t-test (**D**-**H**). Source data and exact p values are provided as a Source Data file.

Supplementary Fig. 11

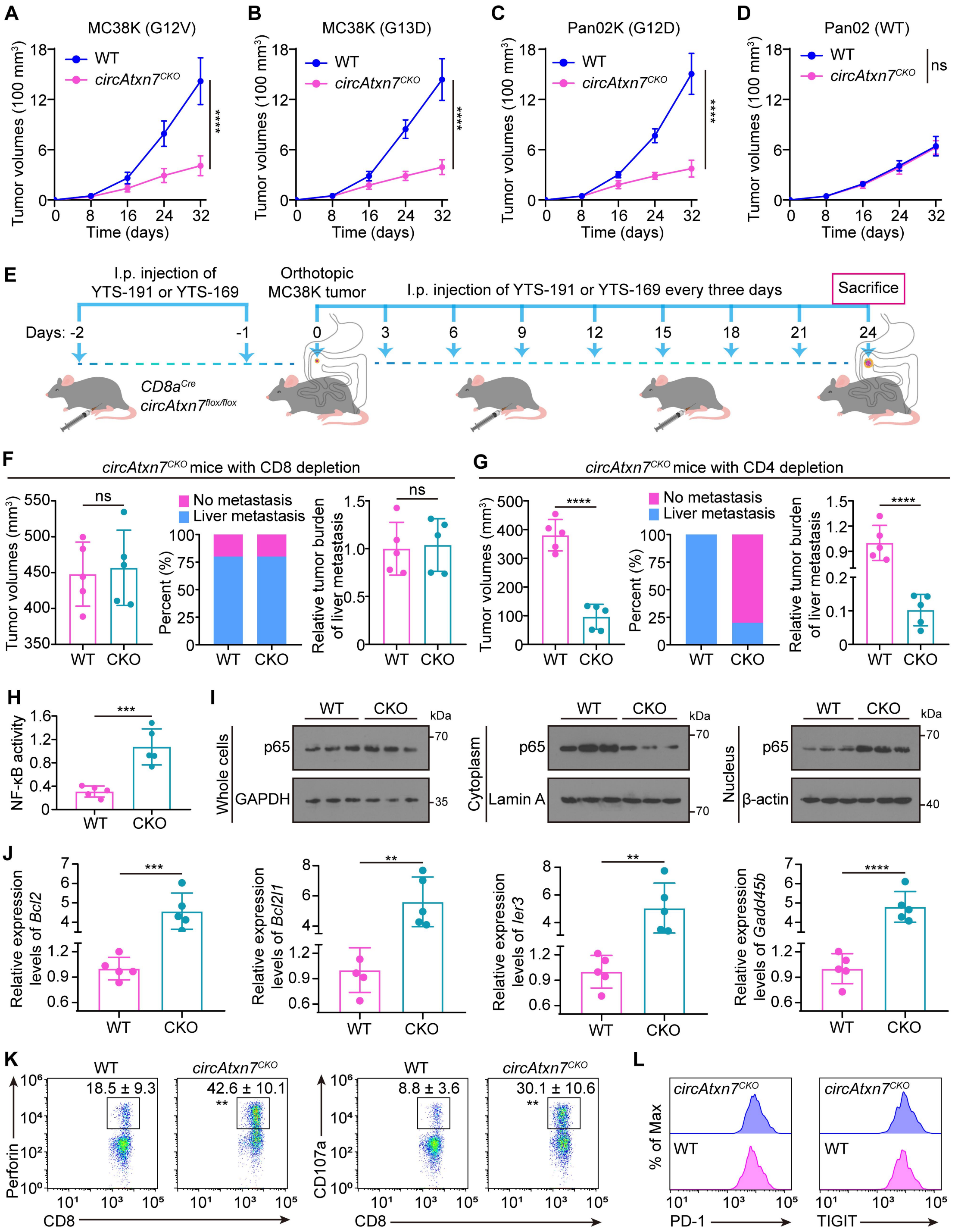

Supplementary Fig. 11 related to Fig. 5. (**A-D**) MC38K (G12V), MC38K (G13D), Pan02K (G12D) or Pan02 (WT) cells were subcutaneously injected into WT or *circAtxn7*^{CKO} mice, and tumor growth was recorded (n = 5 animals). (**E-G**) Before MC38K orthotopic injection, two doses (150 µg/dose) of either YTS-191 or YTS-169 were intraperitoneally injected, followed by eight consecutive infections every three

days. (**F**-**G**) At day 24, CD8- or CD4-depleted MC38K-bearing WT and *circAtxn7*^{CKO} mice were subjected to analyses of tumor volumes, liver metastasis rate, and liver CMV expression (n = 5 samples). (**H-L**) At day 24, $CD8^+$ T cells were purified from MC38K orthotropic xenografts in WT or *circAtxn7*^{CKO} mice, and then subjected to (**H**) NF-κB activity assay (n = 5 samples), (**I**) p65 nuclear translocation (n = 3 samples), (**J**) antiapoptotic gene (Bcl2, Bcl2l1, Ier3, and Gadd45b) expression (n = 5 samples), flow cytometry analysis for (**K**) intracellular perforin and surface marker CD107a (n = 5 samples), and (**L**) exhausted markers PD-1 and TIGIT (n = 5 samples). Numbers (mean ± SD) indicate the percentages of gated cells stained for perforin, CD107a (**K**; *p* values were for comparison with WT). Statistical data presented in this figure show mean values ± SD (**A-D**, **F-H**, **J**). $^{**}p \le 0.01$, $^{***}p \le 0.001$ $^{****}p \le 0.0001$, and ns indicates $p > 0.05$, by two-tailed Student's t-test (**A**-**D**, **F**-**H**, **J**-**K**). Source data and exact p values are provided as a Source Data file.

Supplementary Fig. 12

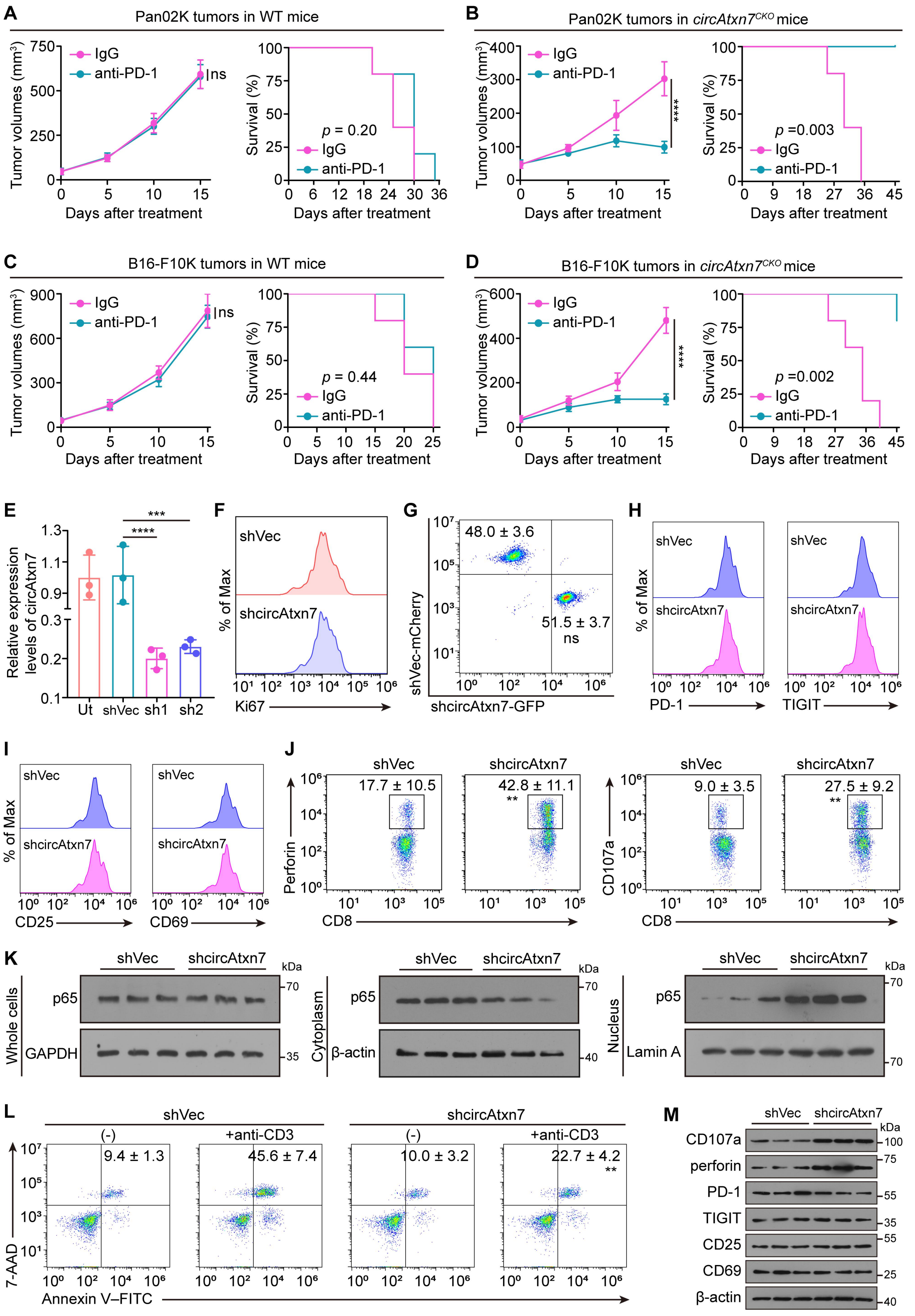

Supplementary Fig. 12 related to Fig. 6. (**A-D**) Tumor growth and survival curves of Pan02K and B16-F10K subcutaneous xenografts in WT (**A**, **C**) or *circAtxn7*^{CKO} (**B**,

D) mice treated with anti-PD-1 antibodies or IgG isotype control antibodies (n = 5 animals). (**E**) circAtxn7 expression level in OT-I cells transduced with lentivirus carrying an expression cassette for circAtxn7 shRNA (sh1 or sh2) or shRNA control vector (shVec) (n = 3 samples). Ut, cells without any treatment. (**F**) Flow cytometric analysis for Ki-67 expression showing no effects of circAtxn7 on OT-I cell proliferation (n = 3 samples). (**G**) Transwell assay-flow cytometry analysis of migrated cells showing no contributions of circAtxn7 to OT-I cell migration (n = 3 samples; *p* values were for comparison with shVec). (**H-I**) Flow cytometric analysis for PD-1, TIGIT, CD107a, CD25, and CD69 showing no effects of circAtxn7 on the exhausted phenotype (**H**) or activation (**I**)of OT-I cells (n = 3 samples). (**J**) Flow cytometric analysis for intracellular perforin and surface marker CD107a showing significant effects of circAtxn7 on the cytotoxic activity of OT-I cells (n = 3 samples). Numbers (mean ± SD) indicate the percentages of gated cells stained for perforin, CD107a (*p* values were for comparison with shVec). (**K**) p65 nuclear translocation of OT-I $CD8^+$ T cells with circAtxn7 silencing or shVec (n = 3 samples). (**L**) Anti-CD3 treatment-induced apoptosis of OT-I cells with circAtxn7 silencing or shVec (n=4 samples). Numerical values (mean ± SD) denote annexin V^+ cell percentages (*p* values were for comparison with shVec). (**M**) Western blots showing the expression of perforin, CD107a PD-1, TIGIT, CD107a, CD25, and CD69 in transferred cells purified from MC38K-OVA tumors (n = 3 samples). Statistical data presented in this figure show mean values ± SD (**A-E**). $^{**}p \leq 0.01$, $^{***}p \leq 0.001$, $^{****}p \leq 0.0001$, and ns indicates $p > 0.05$, by two-tailed Student's t-test (left panels of **A**-**D**, **G**, **J**), one-way ANOVA (**E**, **L**), or two-sided log-rank test (right panels of **A**-**D**). Source data and exact p values are provided as a Source Data file.

Supplementary Fig. 13

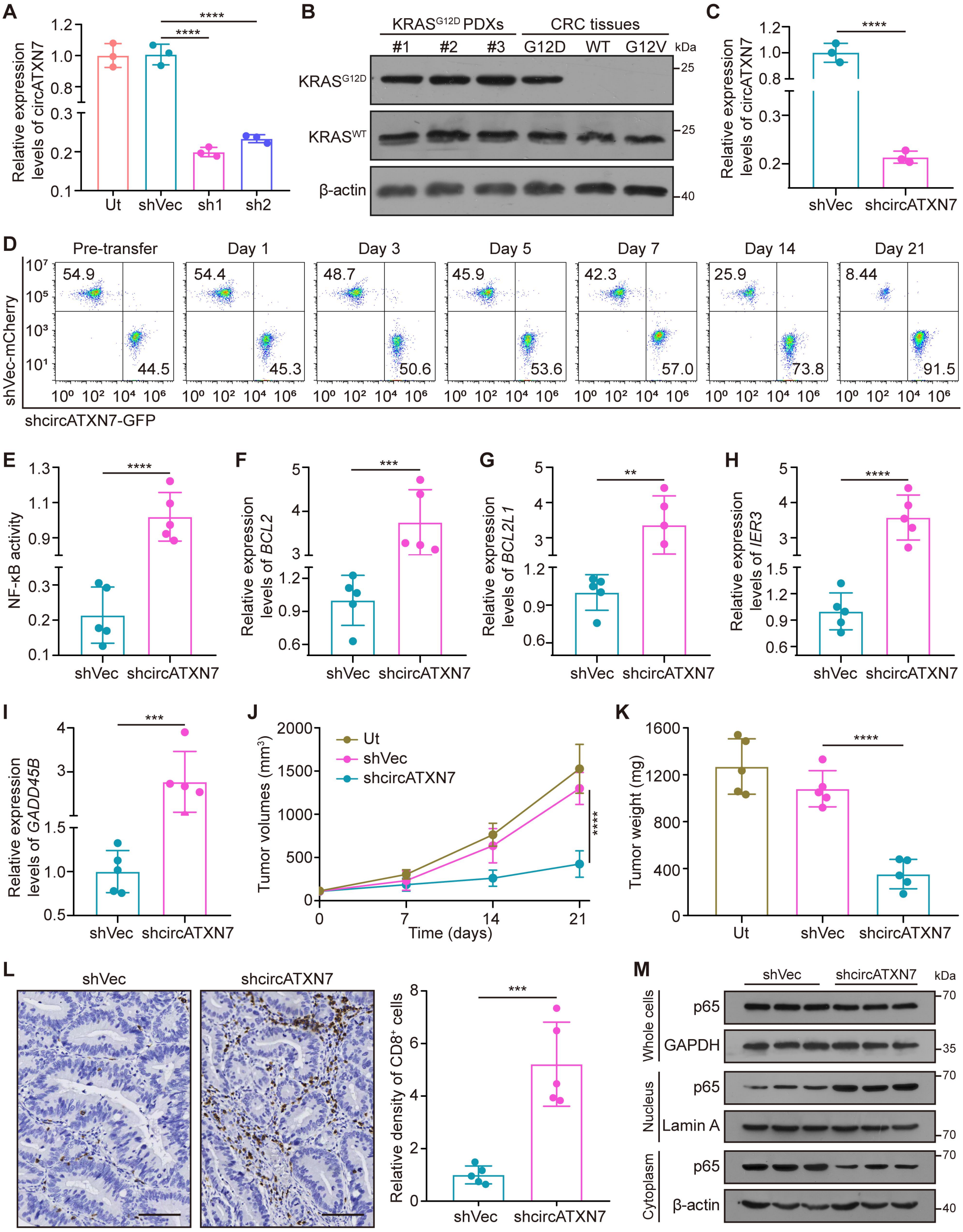

Supplementary Fig. 13 related to Fig. 6. (**A**) circATXN7 expression level in tumor-reactive T cells transduced with lentivirus carrying an expression cassette for circATXN7 shRNA (sh1 or sh2) or shRNA control vector (shVec) (n = 3 independent samples). (**B**) Western blots showing KRAS^{G12D} or KRAS^{WT} expression in CRC PDXs with KRAS^{G12D} and primary CRC tissues with KRAS^{WT} or with indicated

KRAS mutations. Three independent experiments were performed and similar results were obtained. (**C**) qRT-PCR demonstrating circATXN7 silencing still remained in force at the end of ACT experiments (n = 3 independent samples). (**D**) 1.25×10^6 tumor-reactive T cells transduced with GFP-tagged shcircATXN7 and 1.25×10^6 tumor-reactive T cells transduced with mCherry-tagged shVec were mixed and co-injected with 0.5×10^6 antigen-loaded DCs into PDX-bearing mice. Tumors were subjected to flow cytometric analyses of OT-I cell proportion in total $CD8^+$ cells at the indicated time (n = 3 samples). (**E-I**) 2.5×10^6 tumor-reactive $CD8^+$ T cells were transferred into CRC PDXs (n = 5 animals). At day 20 after transfer, tumor-infiltrating $CD8^+$ T cells were purified and then subjected to analyses of NF-κB activity (**E**) and antiapoptotic genes (*BCL2*, *BCL2L1*, *IER3*, and *GADD45B*) expression (**F**-**I**). (**J-M**) Using another PDX model generated from CRC patients with $KRAS^{G13D}$, 2.5×10^6 tumor-reactive $CD8^+$ T cells were transferred into the PDXs. (**J**, n = 5 animals) Tumor growth curves during the course of each indicated treatment. At day 21 after transfer, PDX tumors were subjected to analyses of tumor weights (**K**, n = 5 animals), CD8 staining (**L**, n = 5 animals), and p65 nuclear translocation in tumor-infiltrating $CD8^+$ T cells (**M**, n = 3 animals). Scale bars: 50 µm (**L**). Statistical data presented in this figure show mean values ± SD (**A**, **C**, **E-K**, **L**). $^{**}p \leq 0.01$, $^{***}p \leq 0.001$ and $^{****}p \leq 0.0001$ by one-way ANOVA (**A**, and **J**-**K**) and two-tailed Student's *t*-test (**C**, **E**-**I**, and **L**). Source data and exact p values are provided as a Source Data file.

Supplementary Fig. 14. Gating strategies used for flow cytometry analysis.

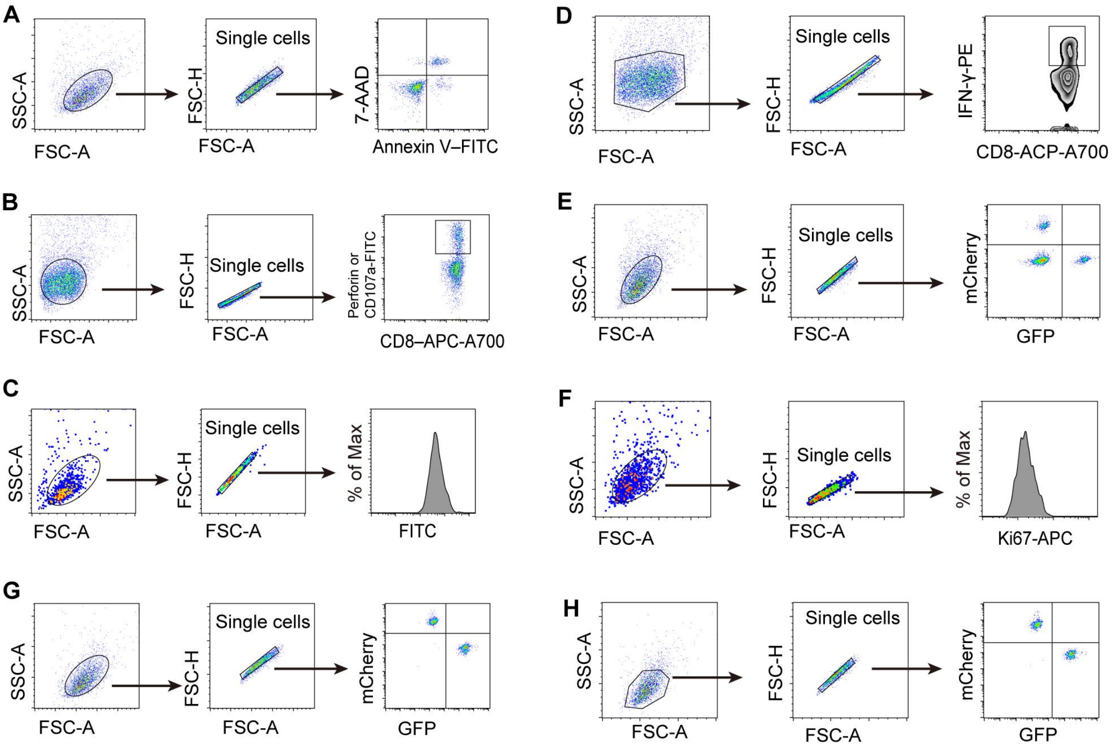

(**A**) Gating strategy for analyzing apoptosis of the indicated cells in Fig. 1A, C-F, H, Fig. 4A, Supplementary Fig. 5B, Supplementary Fig. 12L. (**B**) Gating strategy for analyzing the expression of CD107a or Perforin in Supplementary Fig. 2G, Supplementary Fig. 11K, Supplementary Fig. 12J. (**C**) Gating strategy for analyzing the expression of the surface markers (PD-1, TIGIT, CD25, and CD69) in Supplementary Fig. 2H, Supplementary Fig. 11L, Supplementary Fig. 12H-I. (**D**) Gating strategy for analyzing the IFN-γ expression of CD8 cells in Fig. 5K. (**E**) Gating strategy for analyzing the distribution of transferred cells in Fig. 6D. (**F**) Gating strategy for analyzing Ki67 expression in Supplementary Fig. 5G, Supplementary Fig. 12F. (**G**) Gating strategy for the migratory abilities of the indicated cells in Supplementary Fig. 5H, Supplementary Fig. 12H. (**H**) Gating strategy for analyzing the distribution of transferred cells in Supplementary Fig. 13D.

Supplementary Table 1. NF-κB signaling-associated factors reported in literatures

Factors regulating NF-κB	References
proteins	
TSPAN15	Nat Commun. 2018;9(1):1423[1]
HSPA13	Sci Adv. 2021 Oct 8;7(41):eabh1756[2]
TRIM47	Proc Natl Acad Sci U S A. 2021 Aug 31;118(35):e2100784118[3]
USP12	Cell Death Differ. 2021;28(10):2857-2870[4]
DHX37	Cell. 2019;178(5):1189-1204.e23[5].
DCLK1	Cell Death Differ. 2023;30(5):1184-1197[6]
Dll1	Nat Commun. 2021 Jan 18;12(1):432[7]
CNBP	J Exp Med. 2018;215(12):3136-3150[8]
MALT1	J Clin Invest. 2012;122(12):4698-709[9]
PP4R1	Immunity. 2012;37(4):697-708.[10]
lncRNAs	
TRINGS	EMBO J. 2017 Dec 1;36(23):3483-3500[11]
ASB16-AS1	Gastric Cancer. 2021 Jan;24(1):45-59.[12]
lncRNA-PLACT1	Mol Cancer. 2020;19(1):35[13]
LINC00665	Hepatology. 2020;72(5):1666-1681[14]
PDIA3P1	Hepatology. 2020;71(5):1660-1677[15]
SChLAP1	Clin Cancer Res. 2019;25(22):6868-6881[16]
lncRNA-PCAT1	Nucleic Acids Res. 2019;47(8):4211-4225[17]
CamK-A	Mol Cell. 2018;72(1):71-83.e7[18]
lincRNA-Cox2	Cell Death Differ. 2019;26(1):130-145[19]
NKILA	Cancer Cell. 2015;27(3):370-81[20]
miRNAs	
miR-21	Sci Transl Med. 2021;13(621):eaav7223[21]
miR-29	Blood. 2021;137(18):2481-2494[22]
Mir155	J Clin Invest. 2012;122(11):4190-202.[23]
miR-132	Nucleic Acids Res. 2019;47(7):3580-3593[24]
miR-92a-1-5p	Gut. 2019;68(10):1751-1763[25]
miR-141	Nat Commun. 2018;9(1):5051[26]

miR-378	J Hepatol. 2019;70(1):87-96[27]
miR-150	Blood. 2018;132(22):2389-2400[28]
miR-148a	Cell Death Differ. 2017;24(12):2199-2209[29]
miR-26	Nucleic Acids Res. 2016;44(8):3772-87[30]

Supplementary Table 2. Primers and DNA sequences used in this study

Name	Sequence	Application
Transgene-forward	CAGCAGCAGGTGAGACAAAGT	genotyping of OT-I mice
Transgene-reverse	GGCTTTATAATTAGCTTGGTCC	genotyping of OT-I mice
Internal Positive Control-forward	CAAATGTTGCTTGTCTGGTG	genotyping of OT-I mice
Internal Positive Control-reverse	GTCAGTCGAGTGCACAGTTT	genotyping of OT-I mice
Transgene-forward	CAATGGAAGGAAGTCGTGGT	genotyping of *Cd8a*-Cre mice
Common-reverse	TGGGATTTACAGGGCATACTG	genotyping of *Cd8a*-Cre mice
Wild type-forward	CACACATGCAAGTCTAAATCAG G	genotyping of *Cd8a*-Cre mice
Common-forward	CAATTCAAAGTGATGGGCATAG GAA	genotyping of $circAtxn7^{CKO}$ mice
Common-reverse	TTCCCAGACAGCCAAGGCCATTT AGT	genotyping of $circAtxn7^{CKO}$ mice
CKO-reverse	AGCATACCTTTAGATCACCCTAT CAG	genotyping of $circAtxn7^{CKO}$

		mice
circATXN7-forward	AATCTGTGGGTTGAGGC	qRT-PCR
circATXN7- reverse	GCTCCGACATTCTTTCC	qRT-PCR
circAtxn7- forward (mouse)	GGATGGGACCGAATTGGATGA	qRT-PCR
circAtxn7- reverse (mouse)	CCCGCTCCGACATTCTTTCC	qRT-PCR
Linear-*ATXN7*-forward	CTAGGGGTGGGCTCGTTTC	qRT-PCR
Linear-*ATNX7*-reverse	CCTCGGTACCCCTAGTCCC	qRT-PCR
promoter-*ATXN7*-forward	GCAGATTCGCAACAGGGTG	ChIP-PCR
promoter-*ATNX7*-reverse	ACGCCATTCTGATAGTGGTTGA	ChIP-PCR
BCL2-forward	TGCACCTGACGCCCTTCAC	qRT-PCR
BCL2-reverse	AGACAGCCAGGAGAAATCAAACAG	qRT-PCR
IER3-forward	CCGCACTCCCCAAAAAGAA	qRT-PCR
IER3-reverse	GCTCTCGCGCACCAGGTA	qRT-PCR
GADD45B-forward	ACAGTGGGGGTGTACGAGTC	qRT-PCR
GADD45B-reverse	TTGATGTCGTTGTCACAGCA	qRT-PCR
BCL2L1-forward	CTGCCTCACTTCCTACAAGAGC	qRT-PCR
BCL2L1-reverse	CTGAGGTAGGGAAGACCCTG	qRT-PCR
Bcl2-forward (mouse)	ATGCCTTTGTGGAACTATATGGC	qRT-PCR
Bcl2-reverse (mouse)	GGTATGCACCCAGAGTGATGC	qRT-PCR

Ier3-forward (mouse)	GCTCTGGTCCCGAGATTTTCA	qRT-PCR
Ier3-reverse (mouse)	AGATGATGGCGAACAGGAGAA	qRT-PCR
Gadd45b-forward (mouse)	CAACGCGGTTCAGAAGATGC	qRT-PCR
Gadd45b-reverse (mouse)	GGTCCACATTCATCAGTTTGGC	qRT-PCR
Bcl2l1-forward (mouse)	GACAAGGAGATGCAGGTATTGG	qRT-PCR
Bcl2l1-reverse (mouse)	TCCCGTAGAGATCCACAAAAGT	qRT-PCR
Human β-actin-forward	TCATGAAGTGTGACGTGGACATC	qRT-PCR
Human β-actin-reverse	CAGGAGGAGCAATGATCTTGAT CT	qRT-PCR
18S rRNA-forward	CGGCTACCACATCCAAGGAA	qRT-PCR
18S rRNA-reverse	GCTGGAATTACCGCGGCT	qRT-PCR
GAPDH-forward	CGCTCTCTGCTCCTCCTGTTC	qRT-PCR
GAPDH-reverse	ATCCGTTGACTCCGACCTTCAC	qRT-PCR
CMV-forward	GTCATCGCTATTACCATGGTGAT G	qRT-PCR
	CGG	
CMV-reverse	AGCTCTGCTTATATAGACCTCCC A	qRT-PCR
	CCG	
Mouse β-actin-forward	CCGGCATGTGCAAAGCCGGCTTC G	qRT-PCR
Mouse β-actin-reverse	CTCATTGTAGAAGGTGTGGTGCC	qRT-PCR
sh-circATXN7#2sh-circATXN7#1 or sh-circAtxn7#1 (mouse)	CTGTCGGGAAGGAGCGGAAAGC GGGAAGGAGCGGAAAGAATG	shRNA target siteshRNA

		target site
sh-circAtxn7#2 (mouse)	CTGCCGGGAAGGAGCGGAAAG	shRNA target site
circATXN7 probe	CGACATTCTTTCCGCTCCTTCCC GACAGA	FISH, ISH, RNA pulll down

References

1. Zhang B*, et al.* TSPAN15 interacts with BTRC to promote oesophageal squamous cell carcinoma metastasis via activating NF-κB signaling. *Nature communications* **9**, 1423 (2018).

2. Gao C*, et al.* HSPA13 facilitates NF-κB-mediated transcription and attenuates cell death responses in TNFα signaling. *Science advances* **7**, eabh1756 (2021).

3. Azuma K, Ikeda K, Suzuki T, Aogi K, Horie-Inoue K, Inoue S. TRIM47 activates NF-κB signaling via PKC-ε/PKD3 stabilization and contributes to endocrine therapy resistance in breast cancer. *Proceedings of the National Academy of Sciences of the United States of America* **118**, (2021).

4. Fu Y*, et al.* USP12 promotes CD4 T cell responses through deubiquitinating and stabilizing BCL10. *Cell death and differentiation* **28**, 2857-2870 (2021).

5. Dong M*, et al.* Systematic Immunotherapy Target Discovery Using Genome-Scale In Vivo CRISPR Screens in CD8 T Cells. *Cell* **178**, 1189-1204.e1123 (2019).

6. Luo W*, et al.* Doublecortin-like kinase 1 activates NF-κB to induce inflammatory responses by binding directly to IKKβ. *Cell death and differentiation* **30**, 1184-1197 (2023).

7. Kumar S*, et al.* Dll1 quiescent tumor stem cells drive chemoresistance in breast cancer through NF-κB survival pathway. *Nature communications* **12**, 432 (2021).

8. Chen Y*, et al.* CNBP controls IL-12 gene transcription and Th1 immunity. *The Journal of experimental medicine* **215**, 3136-3150 (2018).

9. Brüstle A*, et al.* The NF-κB regulator MALT1 determines the encephalitogenic potential of Th17 cells. *The Journal of clinical investigation* **122**, 4698-4709 (2012).

10. Brechmann M*, et al.* A PP4 holoenzyme balances physiological and oncogenic nuclear factor-

kappa B signaling in T lymphocytes. *Immunity* **37**, 697-708 (2012).

11. Khan M, Xiang S, Song Z, Wu M. The p53-inducible long noncoding RNA TRINGS protects cancer cells from necrosis under glucose starvation. *The EMBO journal* **36**, 3483-3500 (2017).

12. Fu T, *et al.* ASB16-AS1 up-regulated and phosphorylated TRIM37 to activate NF-κB pathway and promote proliferation, stemness, and cisplatin resistance of gastric cancer. *Gastric cancer : official journal of the International Gastric Cancer Association and the Japanese Gastric Cancer Association* **24**, 45-59 (2021).

13. Ren X, *et al.* lncRNA-PLACT1 sustains activation of NF-κB pathway through a positive feedback loop with IκBα/E2F1 axis in pancreatic cancer. *Molecular cancer* **19**, 35 (2020).

14. Ding J, *et al.* Inflammation-Induced Long Intergenic Noncoding RNA (LINC00665) Increases Malignancy Through Activating the Double-Stranded RNA-Activated Protein Kinase/Nuclear Factor Kappa B Pathway in Hepatocellular Carcinoma. *Hepatology (Baltimore, Md)* **72**, 1666-1681 (2020).

15. Xie C, *et al.* A hMTR4-PDIA3P1-miR-125/124-TRAF6 Regulatory Axis and Its Function in NF kappa B Signaling and Chemoresistance. *Hepatology (Baltimore, Md)* **71**, 1660-1677 (2020).

16. Ji J, *et al.* SChLAP1Long Noncoding RNA Forms a Growth-Promoting Complex with HNRNPL in Human Glioblastoma through Stabilization of ACTN4 and Activation of NF-κB Signaling. *Clinical cancer research : an official journal of the American Association for Cancer Research* **25**, 6868-6881 (2019).

17. Shang Z, *et al.* LncRNA PCAT1 activates AKT and NF-κB signaling in castration-resistant prostate cancer by regulating the PHLPP/FKBP51/IKKα complex. *Nucleic Acids Res* **47**, 4211-4225 (2019).

18. Sang L, *et al.* LncRNA CamK-A Regulates Ca-Signaling-Mediated Tumor Microenvironment Remodeling. *Molecular cell* **72**, 71-83.e77 (2018).

19. Xue Z, *et al.* lincRNA-Cox2 regulates NLRP3 inflammasome and autophagy mediated neuroinflammation. *Cell death and differentiation* **26**, 130-145 (2019).

20. Liu B, *et al.* A cytoplasmic NF-κB interacting long noncoding RNA blocks IκB phosphorylation and suppresses breast cancer metastasis. *Cancer cell* **27**, 370-381 (2015).

21. Kim R, *et al.* A microRNA-21-mediated SATB1/S100A9/NF-κB axis promotes chronic obstructive pulmonary disease pathogenesis. *Science translational medicine* **13**, eaav7223 (2021).

22. Sharma S, *et al.* miR-29 modulates CD40 signaling in chronic lymphocytic leukemia by targeting TRAF4: an axis affected by BCR inhibitors. *Blood* **137**, 2481-2494 (2021).

23. Nazari-Jahantigh M, *et al.* MicroRNA-155 promotes atherosclerosis by repressing Bcl6 in macrophages. *The Journal of clinical investigation* **122**, 4190-4202 (2012).

24. Kong P, *et al.* circ-Sirt1 controls NF-κB activation via sequence-specific interaction and enhancement of SIRT1 expression by binding to miR-132/212 in vascular smooth muscle cells. *Nucleic Acids Res* **47**, 3580-3593 (2019).

25. Li T, *et al.* MicroRNA-92a-1-5p increases CDX2 by targeting FOXD1 in bile acids-induced gastric intestinal metaplasia. *Gut* **68**, 1751-1763 (2019).

26. Ji M, *et al.* Preclinical development of a microRNA-based therapy for intervertebral disc degeneration. *Nature communications* **9**, 5051 (2018).

27. Zhang T, *et al.* MicroRNA-378 promotes hepatic inflammation and fibrosis via modulation of the NF-κB-TNFα pathway. *Journal of hepatology* **70**, 87-96 (2019).

28. Musilova K, *et al.* miR-150 downregulation contributes to the high-grade transformation of follicular lymphoma by upregulating FOXP1 levels. *Blood* **132**, 2389-2400 (2018).

29. Zhu Y, *et al.* miR-148a inhibits colitis and colitis-associated tumorigenesis in mice. *Cell death and differentiation* **24**, 2199-2209 (2017).

30. Chen C, Chang J, Ho Y, Shyu A. MiR-26 down-regulates TNF-α/NF-κB signalling and IL-6 expression by silencing HMGA1 and MALT1. *Nucleic Acids Res* **44**, 3772-3787 (2016).
