# Supplementary material for: Mutant KRAS-activated circATXN7 fosters tumor immunoescape by sensitizing tumor-specific T cells to activation-induced cell death
Source: Nat Commun. 2024 Jan 12;15:499. doi: 10.1038/s41467-024-44779-1 (PMC10786880; doi:10.1038/s41467-024-44779-1)
Supplement: Supplementary file 3 — Reporting Summary [file 41467_2024_44779_MOESM3_ESM.pdf]

nature portfolio

Corresponding author(s): Huashan Liu

Last updated by author(s): Nov 30, 2023

Reporting Summary

Nature Portfolio wishes to improve the reproducibility of the work that we publish. This form provides structure for consistency and transparency in reporting. For further information on Nature Portfolio policies, see our Editorial Policies and the Editorial Policy Checklist.

Statistics

For all statistical analyses, confirm that the following items are present in the figure legend, table legend, main text, or Methods section.

n/a	Confirmed	
☐	☒	The exact sample size (*n*) for each experimental group/condition, given as a discrete number and unit of measurement
☐	☒	A statement on whether measurements were taken from distinct samples or whether the same sample was measured repeatedly
☐	☒	The statistical test(s) used AND whether they are one- or two-sided<br>*Only common tests should be described solely by name; describe more complex techniques in the Methods section.*
☒	☐	A description of all covariates tested
☐	☒	A description of any assumptions or corrections, such as tests of normality and adjustment for multiple comparisons
☐	☒	A full description of the statistical parameters including central tendency (e.g. means) or other basic estimates (e.g. regression coefficient) AND variation (e.g. standard deviation) or associated estimates of uncertainty (e.g. confidence intervals)
☐	☒	For null hypothesis testing, the test statistic (e.g. *F*, *t*, *r*) with confidence intervals, effect sizes, degrees of freedom and *P* value noted<br>*Give P values as exact values whenever suitable.*
☒	☐	For Bayesian analysis, information on the choice of priors and Markov chain Monte Carlo settings
☒	☐	For hierarchical and complex designs, identification of the appropriate level for tests and full reporting of outcomes
☐	☒	Estimates of effect sizes (e.g. Cohen's *d*, Pearson's *r*), indicating how they were calculated

Our web collection on statistics for biologists contains articles on many of the points above.

Software and code

Policy information about availability of computer code

Data collection	Quantitative PCR： Roche LightCycler 480;<br>ELISA: TECAN Infinite 200 PRO;<br>Laser scanning confocal microscopy: Zeiss LSM 780, LSM 800;<br>Flow cytometry: Beckman CytoFLEXFlow cytometer, BD Accuri C6;
Data analysis	Data representation: Graph pad Prism 8.0;<br>Clinical data analysis: Excel 2016, Graph pad Prism 8.0;<br>Quantitative of western blot, chemotactic Assay and fluorescent images: Image J(version 1.43), Excel 2016;<br>Statistical analysis: SPSS 16.0; Graph pad Prism 8.0;<br>Fluorescent images processing: ZEN 2012;<br>Flow cytometry data analysis: FlowJo (versions 7.6 and 10);<br>Gene enrichment analysis: GSEA software (version 4.0.4)

For manuscripts utilizing custom algorithms or software that are central to the research but not yet described in published literature, software must be made available to editors and reviewers. We strongly encourage code deposition in a community repository (e.g. GitHub). See the Nature Portfolio guidelines for submitting code & software for further information.

Data

Policy information about availability of data

All manuscripts must include a data availability statement. This statement should provide the following information, where applicable:

- Accession codes, unique identifiers, or web links for publicly available datasets
- A description of any restrictions on data availability
- For clinical datasets or third party data, please ensure that the statement adheres to our policy

Source data for the circRNA-seq, ChIP-seq, and RNA-seq have been deposited in the Genome Sequence Archive under the accession numbers HRA003320, HRA003223, and CRA007181, respectively. Other data generated in this study are provided in the Supplementary Information/Source Data file. Source data are provided with this paper.

Research involving human participants, their data, or biological material

Policy information about studies with human participants or human data. See also policy information about sex, gender (identity/presentation), and sexual orientation and race, ethnicity and racism.

Reporting on sex and gender	Human CRC samples were obtain from 269 patients with stage I–III CRC in The Sixth Affiliated Hospital of Sun Yat-sen University (97 females, 172 males) and 101 patients with stage IV CRC in Sun Yat-sen University Cancer Center (40 females, 61 males). A total of 87 pancreatic cancer (37 females, 50 males) patients used herein were obtained from Shanghai Outdo Biotech (Shanghai, China). No gender related issues are applied to the analysis.
Reporting on race, ethnicity, or other socially relevant groupings	All the patients included are Chinese people who belong to the yellow race.
Population characteristics	Human cancer samples were obtained from 269 patients with stage I-III colorectal cancers (the maximum age is 86, the minimum age is 23, a median age of 61 years), 101 patients with stage IV colorectal cancers (the maximum age is 79, the minimum age is 26, a median age of 54 years), and 87 pancreatic cancer patients (the maximum age is 77, the minimum age is 33, a median age of 58 years).
Recruitment	Resection material was collected from primary tumors during surgery, which was proven on histopathology. There was no self-selection bias in this study.
Ethics oversight	The approval of the Institutional Review Board of The Sixth Affiliated Hospital of Sun Yat-sen University (Guangzhou, China; approval number: G2020008 and G2022024) and Sun Yat-sen University Cancer Center (Guangzhou, China; approval number: G2021-088-01 and B2022-025-01) covers the collection of formalin-fixed, paraffin-embedded tissues, PBMCs and primary CRC specimens. Informed consent was waived for the collection of formalin-fixed, paraffin-embedded tissues, whereas informed consent was obtained from each patient for the collection of PBMCs and primary CRC specimens. The approval of the Institutional Ethical Review Boards of Shanghai Outdo Biotech (approval number: SHYJS-CP-1901008) covers the collection of formalin-fixed, paraffin-embedded tissues, in which anonymized data were analyzed, and waived the requirement for informed consent.

Note that full information on the approval of the study protocol must also be provided in the manuscript.

Field-specific reporting

Please select the one below that is the best fit for your research. If you are not sure, read the appropriate sections before making your selection.

[x] Life sciences   [ ] Behavioural & social sciences   [ ] Ecological, evolutionary & environmental sciences

For a reference copy of the document with all sections, see nature.com/documents/nr-reporting-summary-flat.pdf

Life sciences study design

All studies must disclose on these points even when the disclosure is negative.

Sample size	No statistical methods were used to predetermine sample size. Yet sample size of animal experiments was estimated on the basis of similar research reported in the literature. In most of the experiments, 3 to 10 mice/samples were sufficient to identify differences between groups with at least 80% power and a 5% significance level. For clinical sample analysis, sample size was determined on the basis of similar research reported in the literature. For in vitro experiments, the sample size was determined based on pilot experiments or previous studies. All experiments included at least 3 independent experiments. The number of independent experiments was indicated in each figure legend.
Data exclusions	No data were excluded.
Replication	For each experiments of the number of biological independent animal/sample/patients is reported in the figure legend. For in vitro experiments at least two-three biological replicates were performed with similar results. For in vivo studies at least 5 animals were allocated per group.

Randomization	The samples used in this study were randomly allocated into control or experimental groups.
Blinding	For in vivo studies, the tumor measurement, treatment and analysis were performed blindly by different researchers. For in vitro studies, cells were treated identically without prior designation.

Behavioural & social sciences study design

All studies must disclose on these points even when the disclosure is negative.

Study description	*Briefly describe the study type including whether data are quantitative, qualitative, or mixed-methods (e.g. qualitative cross-sectional, quantitative experimental, mixed-methods case study).*
Research sample	*State the research sample (e.g. Harvard university undergraduates, villagers in rural India) and provide relevant demographic information (e.g. age, sex) and indicate whether the sample is representative. Provide a rationale for the study sample chosen. For studies involving existing datasets, please describe the dataset and source.*
Sampling strategy	*Describe the sampling procedure (e.g. random, snowball, stratified, convenience). Describe the statistical methods that were used to predetermine sample size OR if no sample-size calculation was performed, describe how sample sizes were chosen and provide a rationale for why these sample sizes are sufficient. For qualitative data, please indicate whether data saturation was considered, and what criteria were used to decide that no further sampling was needed.*
Data collection	*Provide details about the data collection procedure, including the instruments or devices used to record the data (e.g. pen and paper, computer, eye tracker, video or audio equipment) whether anyone was present besides the participant(s) and the researcher, and whether the researcher was blind to experimental condition and/or the study hypothesis during data collection.*
Timing	*Indicate the start and stop dates of data collection. If there is a gap between collection periods, state the dates for each sample cohort.*
Data exclusions	*If no data were excluded from the analyses, state so OR if data were excluded, provide the exact number of exclusions and the rationale behind them, indicating whether exclusion criteria were pre-established.*
Non-participation	*State how many participants dropped out/declined participation and the reason(s) given OR provide response rate OR state that no participants dropped out/declined participation.*
Randomization	*If participants were not allocated into experimental groups, state so OR describe how participants were allocated to groups, and if allocation was not random, describe how covariates were controlled.*

Ecological, evolutionary & environmental sciences study design

All studies must disclose on these points even when the disclosure is negative.

Study description	*Briefly describe the study. For quantitative data include treatment factors and interactions, design structure (e.g. factorial, nested, hierarchical), nature and number of experimental units and replicates.*
Research sample	*Describe the research sample (e.g. a group of tagged Passer domesticus, all Stenocereus thurberi within Organ Pipe Cactus National Monument), and provide a rationale for the sample choice. When relevant, describe the organism taxa, source, sex, age range and any manipulations. State what population the sample is meant to represent when applicable. For studies involving existing datasets, describe the data and its source.*
Sampling strategy	*Note the sampling procedure. Describe the statistical methods that were used to predetermine sample size OR if no sample-size calculation was performed, describe how sample sizes were chosen and provide a rationale for why these sample sizes are sufficient.*
Data collection	*Describe the data collection procedure, including who recorded the data and how.*
Timing and spatial scale	*Indicate the start and stop dates of data collection, noting the frequency and periodicity of sampling and providing a rationale for these choices. If there is a gap between collection periods, state the dates for each sample cohort. Specify the spatial scale from which the data are taken*
Data exclusions	*If no data were excluded from the analyses, state so OR if data were excluded, describe the exclusions and the rationale behind them, indicating whether exclusion criteria were pre-established.*
Reproducibility	*Describe the measures taken to verify the reproducibility of experimental findings. For each experiment, note whether any attempts to repeat the experiment failed OR state that all attempts to repeat the experiment were successful.*
Randomization	*Describe how samples/organisms/participants were allocated into groups. If allocation was not random, describe how covariates were controlled. If this is not relevant to your study, explain why.*
Blinding	*Describe the extent of blinding used during data acquisition and analysis. If blinding was not possible, describe why OR explain why blinding was not relevant to your study.*

Did the study involve field work? ☐ Yes ☐ No

Field work, collection and transport

Field conditions	*Describe the study conditions for field work, providing relevant parameters (e.g. temperature, rainfall).*
Location	*State the location of the sampling or experiment, providing relevant parameters (e.g. latitude and longitude, elevation, water depth).*
Access & import/export	*Describe the efforts you have made to access habitats and to collect and import/export your samples in a responsible manner and in compliance with local, national and international laws, noting any permits that were obtained (give the name of the issuing authority, the date of issue, and any identifying information).*
Disturbance	*Describe any disturbance caused by the study and how it was minimized.*

Reporting for specific materials, systems and methods

We require information from authors about some types of materials, experimental systems and methods used in many studies. Here, indicate whether each material, system or method listed is relevant to your study. If you are not sure if a list item applies to your research, read the appropriate section before selecting a response.

Materials & experimental systems

n/a	Involved in the study	
☐	☒	Antibodies
☐	☒	Eukaryotic cell lines
☒	☐	Palaeontology and archaeology
☐	☒	Animals and other organisms
☒	☐	Clinical data
☒	☐	Dual use research of concern
☒	☐	Plants

Methods

n/a	Involved in the study	
☒	☐	ChIP-seq
☐	☒	Flow cytometry
☒	☐	MRI-based neuroimaging

Antibodies

Antibodies used

All the antibodies are from commercial sources and have been validated by the vendors and their validation data are available on the manufacturer's website. Antibody used for immunoprecipitation (IP), Chromatin immunoprecipitation (ChIP), RNA immunoprecipitation (RIP), immunoblotting (IB), immunofluorescence (IF), blocking/neutralization (BL), immunohistochemistry (IHC) and Flow cytometry (FC) with their respective catalogue number and vendor is mentioned below.

NF-κB p65 (Cell signaling Technology, Cat#8242, Clone: D14E12, IB: 1:1000, IF: 1:400, IP:1:100, RIP: 1:100)
IκBα (Cell signaling Technology, Cat#4814, Clone: L35A5, IB: 1:1000)
Anti-Histone H3 antibody (Abcam, Cat#ab1791, IB: 1:2000)
Anti-beta Actin antibody (Abcam, Cat#ab213262, Clone: EPR21241, IB: 1 µg/ml)
GAPDH (Cell signaling Technology, Cat#2118, Clone: 14C10, IB: 1:1000)
NF-κB1 p105/p50 (Cell signaling Technology, Cat#13586, Clone: D4P4D, IB: 1:1000)
Anti-L-Lactyl Lysine Rabbit pAb (PTM Bio Inc, Cat#PTM-1401RM, IB: 1:500, IP:1:50)
Anti-L-Lactyl-Histone H3 (Lys18) Rabbit mAb (PTM Bio Inc, Cat#PTM-1427RM, IB: 1:500, Chip: 6 µg/5x10e6 cells)
Anti-KRAS antibody (Abcam, Cat#ab275876, Clone: EPR23474-76, IB: 1:1000)
Anti-Ras (mutated G12D) antibody (Abcam, Cat#ab289373, Clone: HL10, IB: 1:1000)
Anti-Lamin A antibody (Abcam, Cat#ab26300, IB: 1 µg/ml)
Anti-human CD8 alpha antibody (Abcam, Cat#ab237709, Clone: CAL66, IHC: 0.25 µg/ml, IF: 1:100, FC: 1:500)
Anti-mouse CD8 alpha antibody (Abcam, Cat#ab217344, Clone: EPR21769, IHC: 1:1000, FC: 1:500)
HLA-A2-CEA Pentamer (ProImmune, Cat#F075-2A-G, A*02:01/YLSGANLNL, FC: 10µg/ml)
Anti-Ataxin 7 antibody (Abcam, Cat#ab259829, Clone: EPR24136-89, IB: 1:1000)
Anti-EpCAM antibody (Abcam, Cat#ab223582, Clone: EPR20532-225, IF: 1:1000)
Anti-human CD4 antibody (Abcam, Cat#ab133616, Clone: EPR6855, IF: 1:100)
Anti-mouse Perforin antibody (Abcam, Cat#ab16074, Clone: CB5.4, IB: 1:800, FC: 1:200)
Anti-human Perforin antibody (Abcam, Cat#ab47225, Clone: B-D48, IB: 1:800, FC: 1:200)
Anti-human CD107a antibody (Abcam, Cat#ab278043, Clone: EPR24395-31, IB: 1:1000, FC: 1:500)
TIGIT Antibody (Thermo Fisher Scientific, Cat#740010M, Clone: MBSA43, FC: 0.25 µg/test)
Anti-mouse CD107a antibody (Abcam, Cat#ab208943, Clone: EPR21026, IB: 1:1000, FC: 1:500)
Anti-human PD1 antibody (Abcam, Cat#ab52587, Clone: NAT105, IB: 1:50, FC: 1:100)
Anti-mouse PD1 antibody (Abcam, Cat#ab300425, Clone: EPR26302-8, IB: 1:1000, FC: 1:500)

Anti-mouse TIGIT Antibody (Thermo Fisher Scientific, Cat#MA5-48199, Clone: 1B4, FC: 1μg/mL)
Anti-human CD3 antibody (BD Bioscience, Cat# 566685, Clone: OKT3, BL: 10μg/ml)
Anti-mouse CD3 antibody (BD Bioscience, Cat# 553057, Clone: 145-2C11 BL: 10μg/ml)
HLA-ABC Monoclonal Antibody (Thermo Fisher Scientific, Cat# MA1-19027, Clone: W6/32, BL: 10μg/ml, FC: 1:50)
TIGIT Antibody (Thermo Fisher Scientific, Cat#PA5-116398, IB: 1:2000)
Anti-Ki67 antibody (Abcam, Cat#ab16667, Clone: SP6, IB: 1:1000, FC: 1:1000)
Anti-CD25 antibody (Abcam, Cat#ab264557, Clone: EPR22816-65, IB: 1:1000, FC: 1:500)
CD69 Antibody (Thermo Fisher Scientific, Cat#MA1-207, Clone: H1.2F3, IB: 1:1000, FC: 1:100)
EpCAM MicroBeads (Miltenyi Biotec, Cat#130-061-101)
CD8 T Cell Isolation Kit (Miltenyi Biotec, Cat#130-096-495)
Anti-PE MicroBeads (Miltenyi Biotec, Cat#130-048-801)
CD8 MicroBeads (Miltenyi Biotec, Cat#130-116-478)
InVivoMAb anti-mouse CD8α (BioXcell, Cat# BE0117, Clone: YTS 169.4)
InVivoMAb anti-mouse CD4 (BioXcell, Cat# BE0003-1, Clone: GK1.5)
InVivoMAb rat IgG2b isotype control (BioXcell, Cat# BE0090, Clone: LTF-2)
InVivoMAb anti-mouse PD-1 (BioXcell, Cat# BE0146, Clone: RMP1-14)
InVivoMAb rat IgG2a isotype control (BioXcell, Cat# BE0089, Clone: 2A3)
IFN gamma Monoclonal Antibody (Thermo Fisher Scientific, Cat#12-7311-82, Clone: XMG1.2, FC: 0.25μg/test)
Anti-mouse CD4 antibody (Abcam, Cat#ab207755, Clone: EPR20122, FC: 1:60)
Anti-CD44 antibody (Abcam, Cat#ab243894, Clone: BLR038F, FC: 1μl/10e6 cells)
Anti-CD62L antibody (Abcam, Cat#ab119834, Clone: MEL-14, FC: 1:1000)
DYKDDDDK Tag (Cell signaling Technology, Cat#14793, Clone: D6W5B, IB: 1:1000)
Anti-rabbit IgG antibody (Cell signaling Technology, Cat#7074, IB: 1:2000)
Anti-mouse IgG antibody (Cell signaling Technology, Cat#7076, IB: 1:2000)

Validation

All antibodies used in this study were obtained from commercial sources and validated according to manufacturers's instruction.

Eukaryotic cell lines

Policy information about cell lines and Sex and Gender in Research

Cell line source(s)

The murine colon carcinoma cell line MC38 were obtained from Kerast Inc. The murine pancreatic adenocarcinoma cell line Pan02 were kindly provided by Prof. Qiongcong Xu (The First Affiliated Hospital, Sun Yat-sen University). The murine melanoma cell line B16F10, and human embryonic kidney 293T (HEK293T) and T2 cells were obtained from ATCC.

Authentication

All the cell lines were authenticated by short tandem repeat profiling prior to use.

Mycoplasma contamination

All the cell lines were tested for mycoplasma contamination by PCR every two months, only mycoplasma negative cells were used for experiments.

Commonly misidentified lines
(See ICLAC register)

No commonly misidentified cell lines were used in this study.

Palaeontology and Archaeology

Specimen provenance

Provide provenance information for specimens and describe permits that were obtained for the work (including the name of the issuing authority, the date of issue, and any identifying information). Permits should encompass collection and, where applicable, export.

Specimen deposition

Indicate where the specimens have been deposited to permit free access by other researchers.

Dating methods

If new dates are provided, describe how they were obtained (e.g. collection, storage, sample pretreatment and measurement), where they were obtained (i.e. lab name), the calibration program and the protocol for quality assurance OR state that no new dates are provided.

☐ Tick this box to confirm that the raw and calibrated dates are available in the paper or in Supplementary Information.

Ethics oversight

Identify the organization(s) that approved or provided guidance on the study protocol, OR state that no ethical approval or guidance was required and explain why not.

Note that full information on the approval of the study protocol must also be provided in the manuscript.

Animals and other research organisms

Policy information about studies involving animals; ARRIVE guidelines recommended for reporting animal research, and Sex and Gender in Research

Laboratory animals	Mus musculus, age 6-8 weeks was used for the experiments.CircAtxn7loxp/loxp mice were generated using CRISPR-Cas9-mediated genome editing by Cyagen Biosciences Inc. (China). Immunocompromised NOD.SCID mice were obtained from GemPharmatech (China) and used to establish patient-derived xenograft models. OT-I (C57BL/6-Tg (TcraTcrb)1100Mjb/J), Cd8a-Cre (C57BL/6-Tg (Cd8a-cre)1Itan/J). To generate mice with circAtxn7 conditional deletion in CD8+ T cells, CircAtxn7loxp/loxp mice were crossed with Cd8a-Cre mice.
Wild animals	No wild animals were used in the study.
Reporting on sex	The phenotypes were observed indiscriminately in male and female mice. No gender related issues are applied to this work
Field-collected samples	No filed-collected samples were used for this study.
Ethics oversight	All animal work was done under the protocols approved by the Institutional Animal Care and Use Committee (IACUC), Sun Yat-sen University (approval number: SYSU-IACUC-2020-000438, SYSU-IACUC-2021-000285 and SYSU-IACUC-2021-000642).

Note that full information on the approval of the study protocol must also be provided in the manuscript.

Clinical data

Policy information about clinical studies

All manuscripts should comply with the ICMJEguidelines for publication of clinical research and a completedCONSORT checklist must be included with all submissions.

Clinical trial registration	*Provide the trial registration number from ClinicalTrials.gov or an equivalent agency.*
Study protocol	*Note where the full trial protocol can be accessed OR if not available, explain why.*
Data collection	*Describe the settings and locales of data collection, noting the time periods of recruitment and data collection.*
Outcomes	*Describe how you pre-defined primary and secondary outcome measures and how you assessed these measures.*

Dual use research of concern

Policy information about dual use research of concern

Hazards

Could the accidental, deliberate or reckless misuse of agents or technologies generated in the work, or the application of information presented in the manuscript, pose a threat to:

No	Yes	
☐	☐	Public health
☐	☐	National security
☐	☐	Crops and/or livestock
☐	☐	Ecosystems
☐	☐	Any other significant area

Experiments of concern

Does the work involve any of these experiments of concern:

No	Yes	
☐	☐	Demonstrate how to render a vaccine ineffective
☐	☐	Confer resistance to therapeutically useful antibiotics or antiviral agents
☐	☐	Enhance the virulence of a pathogen or render a nonpathogen virulent
☐	☐	Increase transmissibility of a pathogen
☐	☐	Alter the host range of a pathogen
☐	☐	Enable evasion of diagnostic/detection modalities
☐	☐	Enable the weaponization of a biological agent or toxin
☐	☐	Any other potentially harmful combination of experiments and agents

Plants

Seed stocks	NA.
Novel plant genotypes	NA.
Authentication	NA.

ChIP-seq

Data deposition

☒ Confirm that both raw and final processed data have been deposited in a public database such as GEO.

☒ Confirm that you have deposited or provided access to graph files (e.g. BED files) for the called peaks.

Data access links<br>*May remain private before publication.*	https://bigd.big.ac.cn/gsa-human/browse/HRA003223
Files in database submission	HRR777326_f1.fastq.gz; HRR777326_r2.fastq.gz; HRR777327_f1.fastq.gz; HRR777327_r2.fastq.gz
Genome browser session<br>(e.g. UCSC)	NA

Methodology

Replicates	*Describe the experimental replicates, specifying number, type and replicate agreement.*
Sequencing depth	*Describe the sequencing depth for each experiment, providing the total number of reads, uniquely mapped reads, length of reads and whether they were paired- or single-end.*
Antibodies	*Describe the antibodies used for the ChIP-seq experiments; as applicable, provide supplier name, catalog number, clone name, and lot number.*
Peak calling parameters	*Specify the command line program and parameters used for read mapping and peak calling, including the ChIP, control and index files used.*
Data quality	*Describe the methods used to ensure data quality in full detail, including how many peaks are at FDR 5% and above 5-fold enrichment.*
Software	*Describe the software used to collect and analyze the ChIP-seq data. For custom code that has been deposited into a community repository, provide accession details.*

Flow Cytometry

Plots

Confirm that:

- [x] The axis labels state the marker and fluorochrome used (e.g. CD4-FITC).
- [x] The axis scales are clearly visible. Include numbers along axes only for bottom left plot of group (a 'group' is an analysis of identical markers).
- [x] All plots are contour plots with outliers or pseudocolor plots.
- [x] A numerical value for number of cells or percentage (with statistics) is provided.

Methodology

Sample preparation	Human peripheral blood mononuclear cells (PBMCs) were donated by healthy donors or CRC patients, and isolated using Ficoll-Paque (GE Healthcare) according to the manufacturer's instructions. CD8+ T-cells were obtained from PBMCs using CD8 + T cell Isolation Kits (Miltenyi Biotec). Tumor-specific CTLs were purified from freshly resected tumor samples of HLA-A2+ patients with CRC expressing CEA using anti-CEA Pentamer-PE (YLSGANLNL; ProImmune), respectively. OT-I cells were acquired with the spleens from transgenic OT-I expressing mice using CD8+ T cell Isolation Kits (Miltenyi Biotec).
Instrument	Beckman CytoFLEXFlow cytometer, BD Accuri C6
Software	FlowJo (versions 7.6, versions 10)
Cell population abundance	Purity of FACS-sorted samples was analysed by flow cytometry. Purity of the samples was >90%.
Gating strategy	Starting cells were gated by FSC/SSC gates. Gates indicating boundaries between "positive" and "negative" are according to the isotype staining. Expression of indicated proteins were checked on these populations as indicated in the figures and figure legends

- [x] Tick this box to confirm that a figure exemplifying the gating strategy is provided in the Supplementary Information.

Magnetic resonance imaging

Experimental design

Design type	*Indicate task or resting state; event-related or block design.*
Design specifications	*Specify the number of blocks, trials or experimental units per session and/or subject, and specify the length of each trial or block (if trials are blocked) and interval between trials.*
Behavioral performance measures	*State number and/or type of variables recorded (e.g. correct button press, response time) and what statistics were used to establish that the subjects were performing the task as expected (e.g. mean, range, and/or standard deviation across subjects).*

Acquisition

Imaging type(s)	*Specify: functional, structural, diffusion, perfusion.*
Field strength	*Specify in Tesla*
Sequence & imaging parameters	*Specify the pulse sequence type (gradient echo, spin echo, etc.), imaging type (EPI, spiral, etc.), field of view, matrix size, slice thickness, orientation and TE/TR/flip angle.*
Area of acquisition	*State whether a whole brain scan was used OR define the area of acquisition, describing how the region was determined.*

Diffusion MRI ☐ Used ☐ Not used

Preprocessing

Preprocessing software	*Provide detail on software version and revision number and on specific parameters (model/functions, brain extraction, segmentation, smoothing kernel size, etc.).*
Normalization	*If data were normalized/standardized, describe the approach(es): specify linear or non-linear and define image types used for transformation OR indicate that data were not normalized and explain rationale for lack of normalization.*
Normalization template	*Describe the template used for normalization/transformation, specifying subject space or group standardized space (e.g. original Talairach, MNI305, ICBM152) OR indicate that the data were not normalized.*

Noise and artifact removal | *Describe your procedure(s) for artifact and structured noise removal, specifying motion parameters, tissue signals and physiological signals (heart rate, respiration).*

Volume censoring | *Define your software and/or method and criteria for volume censoring, and state the extent of such censoring.*

Statistical modeling & inference

Model type and settings | *Specify type (mass univariate, multivariate, RSA, predictive, etc.) and describe essential details of the model at the first and second levels (e.g. fixed, random or mixed effects; drift or auto-correlation).*

Effect(s) tested | *Define precise effect in terms of the task or stimulus conditions instead of psychological concepts and indicate whether ANOVA or factorial designs were used.*

Specify type of analysis: ☐ Whole brain ☐ ROI-based ☐ Both

Statistic type for inference | *Specify voxel-wise or cluster-wise and report all relevant parameters for cluster-wise methods.*

(See Eklund et al. 2016)

Correction | *Describe the type of correction and how it is obtained for multiple comparisons (e.g. FWE, FDR, permutation or Monte Carlo).*

Models & analysis

n/a	Involved in the study
☐	☐ Functional and/or effective connectivity
☐	☐ Graph analysis
☐	☐ Multivariate modeling or predictive analysis

Functional and/or effective connectivity | *Report the measures of dependence used and the model details (e.g. Pearson correlation, partial correlation, mutual information).*

Graph analysis | *Report the dependent variable and connectivity measure, specifying weighted graph or binarized graph, subject- or group-level, and the global and/or node summaries used (e.g. clustering coefficient, efficiency, etc.).*

Multivariate modeling and predictive analysis | *Specify independent variables, features extraction and dimension reduction, model, training and evaluation metrics.*
